# Real-space investigation of polarons in hematite Fe_2_O_3_

**DOI:** 10.1126/sciadv.adp7833

**Published:** 2024-11-01

**Authors:** Jesus Redondo, Michele Reticcioli, Vit Gabriel, Dominik Wrana, Florian Ellinger, Michele Riva, Giada Franceschi, Erik Rheinfrank, Igor Sokolović, Zdenek Jakub, Florian Kraushofer, Aji Alexander, Eduard Belas, Laerte L. Patera, Jascha Repp, Michael Schmid, Ulrike Diebold, Gareth S. Parkinson, Cesare Franchini, Pavel Kocan, Martin Setvin

**Affiliations:** ^1^ Department of Surface and Plasma Science, Faculty of Mathematics and Physics, Charles University, 180 00 Prague, Czech Republic.; ^2^ Institute of Applied Physics, TU Wien, 1040 Vienna, Austria.; ^3^ Institute of Physics, Czech Academy of Sciences, Cukrovarnická 10, 162 00 Prague 6, Czech Republic.; ^4^ University of Vienna, Faculty of Physics, Center for Computational Materials Science, Vienna, Austria.; ^5^ Marian Smoluchowski Institute of Physics, Jagiellonian University, 30-348 Krakow, Poland.; ^6^ Institute of Experimental and Applied Physics, University of Regensburg, 93040 Regensburg, Germany.; ^7^ Institute of Physical Chemistry, University of Innsbruck, 6020 Innsbruck, Austria.; ^8^ Dipartimento di Fisica e Astronomia, Università di Bologna, 40127 Bologna, Italy.

## Abstract

In polarizable materials, electronic charge carriers interact with the surrounding ions, leading to quasiparticle behavior. The resulting polarons play a central role in many materials properties including electrical transport, interaction with light, surface reactivity, and magnetoresistance, and polarons are typically investigated indirectly through these macroscopic characteristics. Here, noncontact atomic force microscopy (nc-AFM) is used to directly image polarons in Fe_2_O_3_ at the single quasiparticle limit. A combination of Kelvin probe force microscopy (KPFM) and kinetic Monte Carlo (KMC) simulations shows that the mobility of electron polarons can be markedly increased by Ti doping. Density functional theory (DFT) calculations indicate that a transition from polaronic to metastable free-carrier states can play a key role in migration of electron polarons. In contrast, hole polarons are significantly less mobile, and their hopping is hampered further by trapping centers.

## Teaser

Polarons (electrons trapped in the crystal lattice) were imaged in real space by noncontact atomic force microscopy.

## INTRODUCTION

Hematite has long been touted as a promising photoanode for photocatalytic water splitting due to its ≈ 2-eV bandgap, but its practical use is hindered by sluggish reaction kinetics (*1*–*3*). This is partly because photogenerated electrons and holes interact with the crystal lattice and form localized, self-trapped quasiparticles known as polarons (*4*, *5*) that have a major impact on macroscopic materials properties (*6*–*13*). Polarons show a lower mobility than free charge carriers because they can only diffuse together with their own polarization cloud of lattice distortions.

The photocatalytic performance of hematite is often enhanced by doping, which increases the polaron concentration and, seemingly, their mobility (*3*, *14*, *15*). This is counterintuitive, however, because defects usually impede electron transport in materials. Similar mobility enhancement has been reported for other materials including CuO:Li (*16*), NiO (*17*), or BiVO_4_ (*18*). The current experimental studies lack information about the behavior of excess charges at the atomic level, however. Here, we provide this information using a combination of noncontact atomic force microscopy (nc-AFM) and density functional theory (DFT) calculations.

## RESULTS AND DISCUSSION

**Fig. 1.**
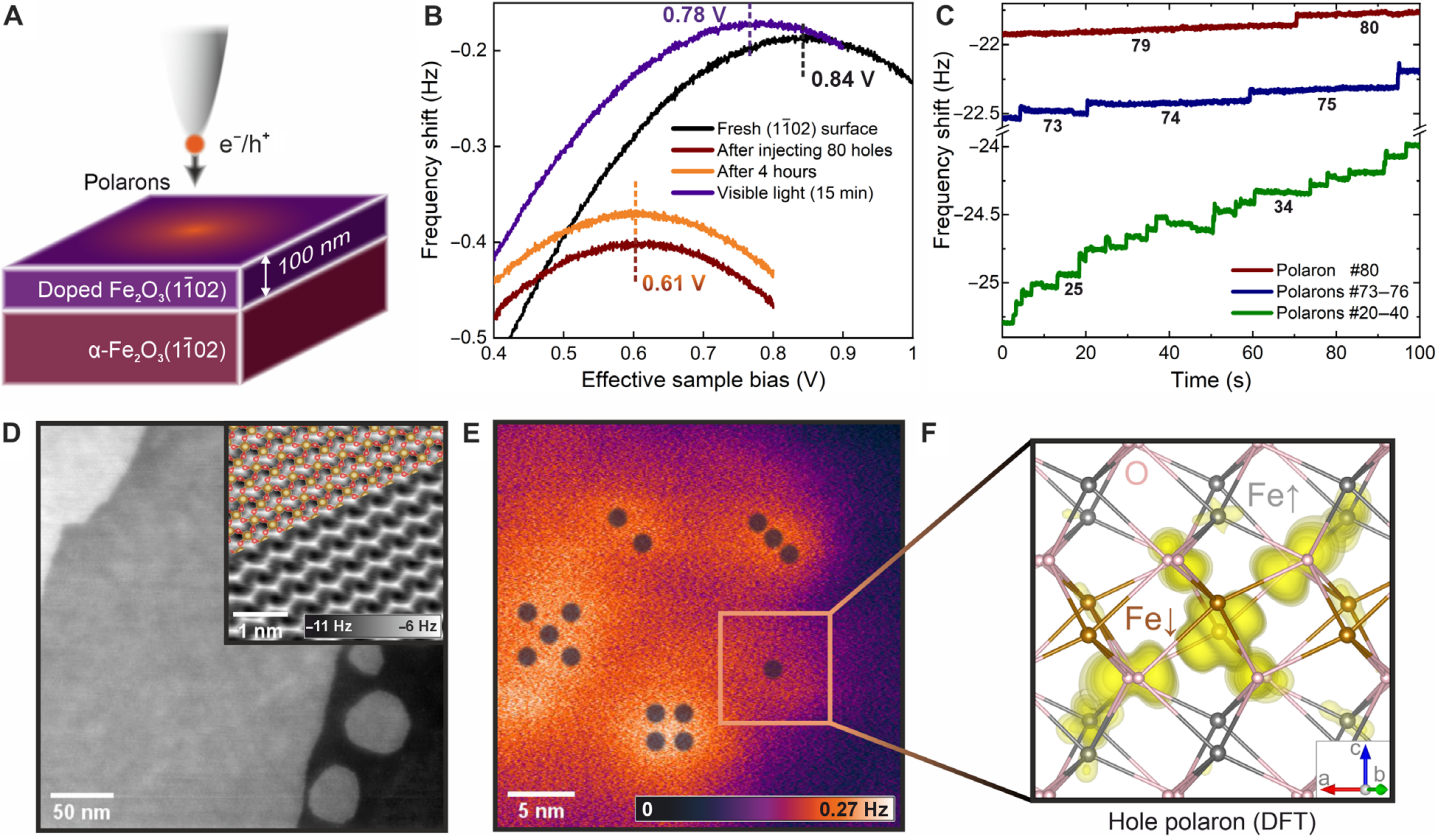
Charge injection in hematite. (**A**) Sketch of the experimental setup. (**B**) Kelvin parabolas showing the LCPD of the pristine surface (black) after injecting ~80 holes (red) into a 0.1% Ni-doped surface, after waiting for 4 hours (orange), and after illuminating the surface by visible light (violet). (**C**) Time dependence of the frequency shift when injecting holes into the surface at effective *V*_S_ = −1.2 V; each jump indicates formation of one individual hole polaron. (**D**) Topographic noncontact (nc)-AFM image of the surface obtained at a constant detuning of −0.57 Hz, *A* = 600 pm, and *V*_S_ = 80 mV. The inset shows an atomically resolved constant-height nc-AFM image, overlaid with the structural model. (**E**) Map of electrostatic forces induced by various numbers of hole polarons injected into the surface at locations marked by the respective numbers of dots. (**F**) Calculated charge densities of a single hole polaron in bulk hematite. O atoms are shown in pink and Fe in brown and gray to distinguish between opposite orientations of the local magnetic moments. All experimental data were measured at *T* = 4.7 K.

The possibility of imaging and manipulating single polarons in real space is demonstrated in Fig. 1 using hole polarons. The experiments were performed on the (1 − 102) surface of Fe_2_O_3_, a nonpolar face that preferably terminates with the surface in a simple (1 × 1) configuration (*19*, *20*). Thin films (50 to 100 nm) of differently doped hematite were grown (*21*) on natural Fe_2_O_3_ single-crystal substrates (see the sketch in Fig. 1A). The substrates are nominally undoped; sample treatment renders them slightly n-type. Electron and hole polarons have been studied on *n*-doped films [Ti doping with concentrations 0, 0.03, 0.7, and 3 atomic % (at %)] and *p*-doped (0.1 at % Ni), respectively (*22*, *23*).

An example of the surface topography is shown in Fig. 1D, with more details discussed in fig. S1. Migration of polarons is frozen out in the lattice at cryogenic temperatures; therefore, the material can be locally charged with an atomic force microscopy (AFM) (*24*) tip, as illustrated in Fig. 1B. Excess electrons or holes can be injected from the tip when a positive or negative bias is applied to the sample, respectively, while the tip is in tunneling contact (≲1 nm distance). Kelvin parabolas (*25*) [where maxima correspond to the local contact potential difference (LCPD)] yield the electrostatic potential above the surface, provided that the tip-sample distance is large enough to prevent electron exchange by tunneling (≳3 nm). The black and red parabolas demonstrate how the LCPD changed after injecting ~80 holes into the surface by tunneling with a negative sample bias. The resulting hole polarons are stable for many hours, see the orange parabola in Fig. 1B. The small vertical shift is attributed to thermal drift. A characteristic feature of polarons is that their mobility can be promoted by light or thermal excitations (unlike for structural defects). The effect of light is demonstrated by the violet parabola, where the LCPD approaches its initial value upon illuminating the sample by visible light (regular room illumination; note that hematite has a bandgap within the visible region, 1.9 to 2.4 eV) (*1*, *20*).

The process of charge injection can be studied with single-quasiparticle precision, as illustrated in Fig. 1C for hole polarons. The tip was positioned ~0.5 nm from the surface, and an effective bias voltage of −1.2 V was applied to the *p*-doped sample, resulting in the formation of hole polarons. The frequency shift shows stepwise increases. It is established that these steps correspond to single-electron charging events (*26*–*28*). Here, we attribute it to the tunneling of single holes into the region below the tip. All the newly formed polarons appear to be created very close to the tip apex because all the steps have a comparable magnitude. As more and more polarons are injected, further tunneling is suppressed due to the electrostatic potential of the so-far injected charges (*29*) that accumulate in the region under the tip. This is illustrated by plotting different sections of the injection curve in Fig. 1C. The cloud of polarons relaxes during the injection process, as evidenced by the apparent drift of the measured frequency shift in between some injection events. These relaxations are promoted by Coulomb repulsions among polarons and the electric field of the tip. More details and analogous curves for electron injection are provided in fig. S3.

Figure 1E shows that single hole polarons can be placed at specific positions. Different numbers of holes (one to five) have been injected in locations marked by the respective numbers of dots. The image in Fig. 1E is a difference of constant height Δ*f* (frequency shift) images measured at identical parameters before and after the polaron injection; full details of this experiment are given in figs. S4 and S5. Areas with increasing brightness form for one to three polarons. For higher numbers, the quasiparticles start to spread due to repulsive Coulomb interactions. Note that the apparent spot size of ~5 nm is attributed to the effective size of the tip apex; the calculated hole polaron size is approximately 0.7 nm, see Fig. 1F. The hole is localized mostly on a single Fe atom (33%), strongly hybridizing with the surrounding oxygen atoms (*29*) (the corresponding density of states is shown in fig. S11). We note that the tip-sample distance for measuring Fig. 1E was approximately 2 nm. Any attempts for a closer approach and imaging the area with atomic resolution resulted in injecting and manipulating polarons due to the overlap of the tip and surface wave functions.

**Fig. 2.**
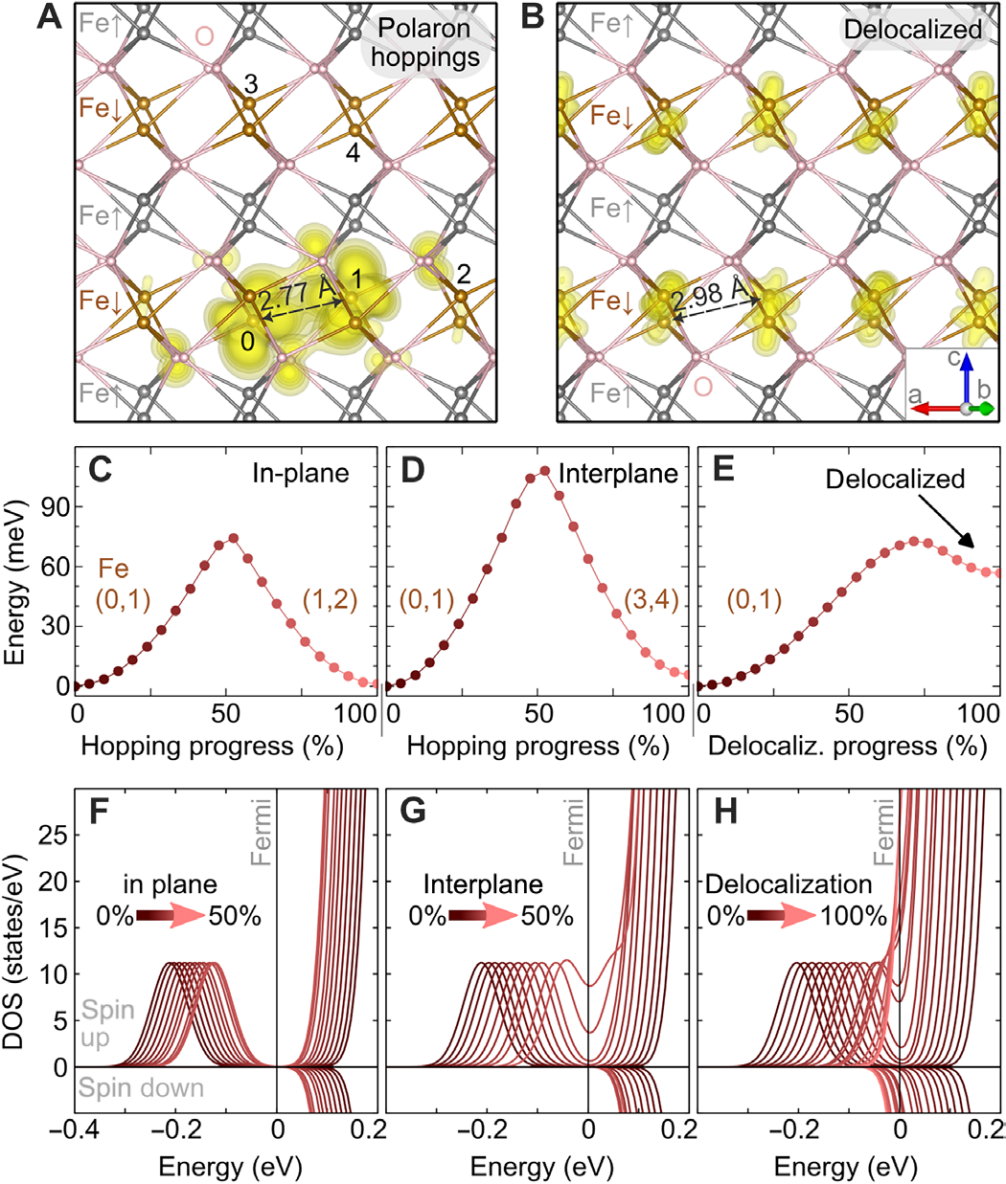
Electron-polaron migration in hybrid-functional DFT. (**A**) The isodensity of an electron polaron in bulk hematite Fe_2_O_3_. At the initial state, the excess electron is mostly localized on Fe atoms marked 0 and 1. The polaron can migrate within the plane of Fe atoms with the same spin (in-plane hop) to atoms 1,2 or across the plane with the opposite spin (inter-plane hop) to atoms 3,4. O atoms are shown in pink, and Fe in brown and gray to distinguish between opposite orientations of the local magnetic moments. (**B**) Spatial distribution of a delocalized electron; the charge in the lowest (partially) occupied band at the bottom of the conduction band is shown. Panels (**C**) to (**E**) show the energy barriers as a function of the progressing lattice distortion for in-plane (C) and interplane (D) polaron hopping, as well as for the delocalization process (E). Panels (**F**) to (**H**) show the evolution of the spin-resolved density of states during the diffusion processes in the top panels.

The key challenge in polaron physics is understanding their complex kinetics. We first discuss the possible mechanisms of migration, considering electron polarons (the more mobile species compared to hole polarons). Figure 2 shows results of hybrid functional DFT calculations with one excess electron in the slab. The projection of the electron polaron states into the local atomic orbitals is plotted in Fig. 2A; two Fe atoms equally share 80% of the electron charge. These two Fe atoms show the typical d^5^ occupation of the spin majority channel in the antiferromagnetically ordered lattice, and the excess electron occupies the minority spin channel (see sketch in fig. S11). The antiferromagnetic ordering of hematite is widely assumed to have a marked impact on the isotropy of polaron migration (see Fig. 2, C and D) (*29*–*31*). The calculated barriers for migration within and across the (0001) planes are very different, 75 and 110 meV, respectively. The anisotropy stems from the fact that the spins in the adjacent Fe layers are opposite. The interplane transition leads through Fe atoms with no available states in the spin channel of the excess electron. However, analysis of the density of states during the hopping shows an atypical behavior: For in-plane hopping, the polaronic state remains below the bottom of the conduction band (Fig. 2F), while the intermediate states of the interplane hopping overlap with the conduction band (Fig. 2G). This indicates a possibility of a transport mechanism based on the temporary delocalization of the polaronic state (*32*).

We modeled the system with the excess electron occupying the bottom of the conduction band, see the delocalized free-electron solution in Fig. 2B. The barrier for delocalizing the electron is 70 meV only (Fig. 2E comparable to the nearest neighbor hopping in Fig. 2C. This indicates a close competition between the nearest neighbor hopping and a mechanism known as the random flight (*33*, *34*): The polaron gets destabilized by thermal distortions, and the resulting free electron–like transition state then relocalizes at another available Fe site.

**Fig. 3.**
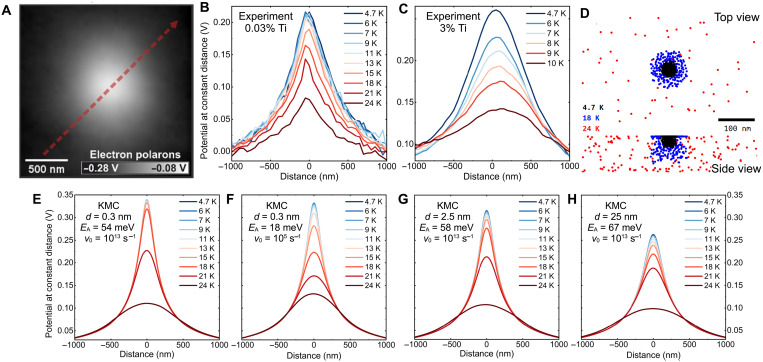
Thermally activated diffusion of electron-polarons. (**A**) Map of LCPD induced by ~300 electron polarons injected into the hematite surface doped with 3% Ti. (**B** and **C**) Effective LCPD line profiles across a cloud of ~300 electron polarons, measured after gradual annealing to increasing temperatures. Ti doping levels were 0.03 and 3%, respectively. The path of the line profile is denoted by the dashed red arrow in (A). (**D**) Evolution of the electron-polaron cloud during gradual annealing, as simulated by KMC, mimicking the experimental procedure of annealing for 10 min at each temperature, using the model in (G). (**E** to **H**) KMC simulations of the profiles in (B) using various parameters of hopping distance (*d*), activation energy (*E*_A_), and frequency prefactor (ν_0_) (the values are in each plot).

**Fig. 4.**
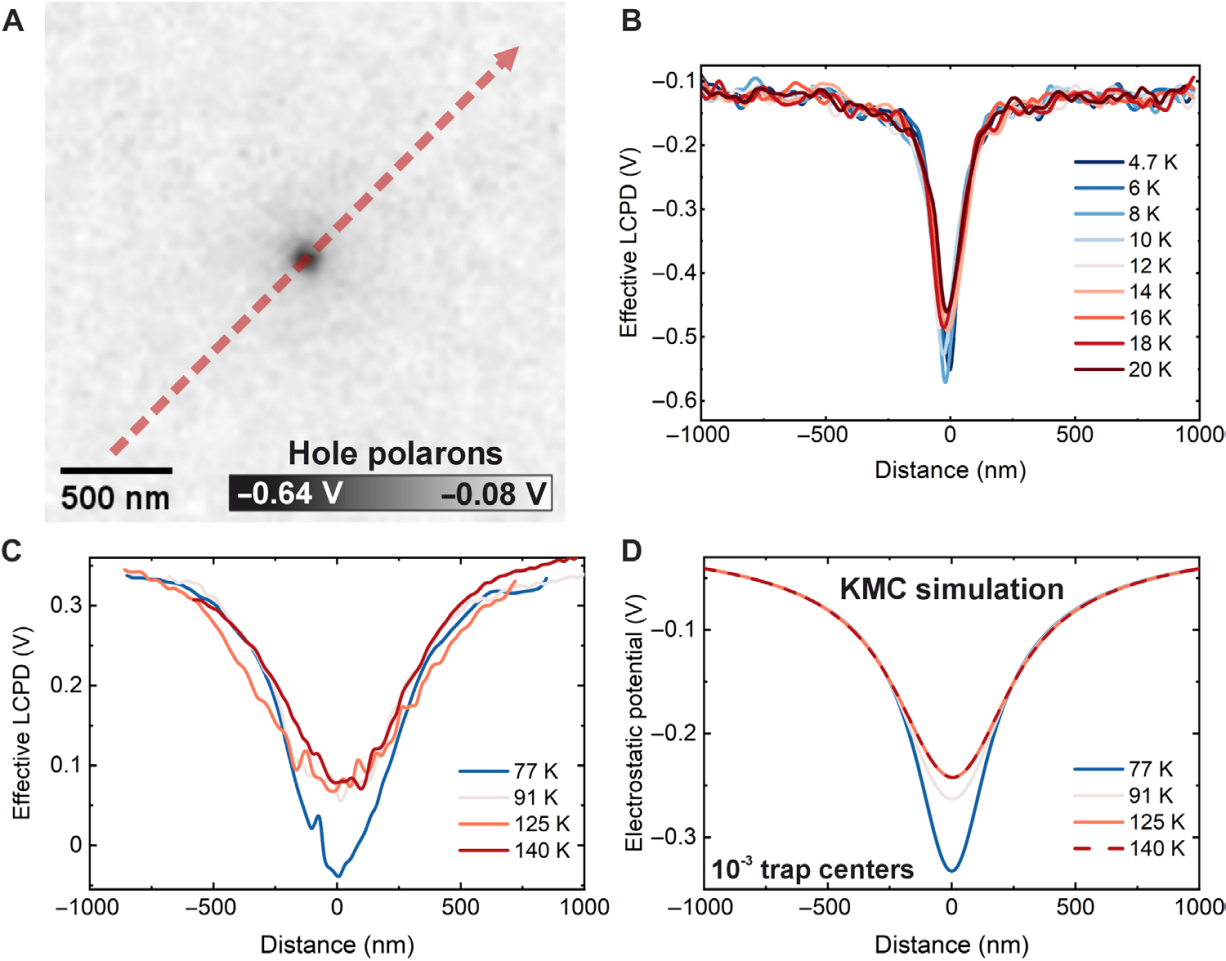
Diffusion of hole polarons. Measured in a p-doped film (0.1% Ni). (**A**) Map of LCPD induced by ~230 hole polarons injected into the surface at *T* = 4.7 K. (**B** and **C**) Effective LCPD from a cloud of ~300 and 150 hole polarons, respectively, and its evolution upon annealing up to 20 and 140 K, respectively. The clouds were injected at *T* = 4.7 K and 77 K, respectively. (**D**) KMC simulation of the hole diffusion denoted in (C), with introduction of hole-trapping centers with a concentration of 10^−3^. For other trap concentrations, see fig. S10.

The experimental results on mobility of electron and hole polarons are summarized in Figs. 3 and 4, respectively. We have used statistical sets of many (approximately hundreds) charge carriers injected into the surface (*35*). This approach allows us to neglect the influence of tip-induced polaron manipulations. The spatial outreach of these injected polaron clouds is shown in the LCPD maps in Figs. 3A and 4A. Note that these images are micrometers wide. The cloud of holes (Fig. 4A) is more confined in space compared to electrons (Fig. 3A) due to the lower mobility of hole polarons (*29*).

In Fig. 3, each experiment began by injecting ~300 electrons and measuring an LCPD map. Subsequently, the sample was gradually annealed to increasing temperatures. In each annealing cycle, the temperature was kept at the given value for 10 min and decreased back to 4.7 K for imaging. The cloud evolves when the polaron mobility is enhanced by thermal excitations. Line profiles across the cloud are shown in Fig. 3 (B and C). The clouds always maintain circular symmetry. The temperature dependence of the cloud profile shows that the dominant effects responsible for polaron migration are thermal excitations combined with electrostatic interactions among the polarons. The stability of these polaron clouds depends critically on doping: Repeating the same experiment for several doping levels of Ti (see Fig. 3, B and C, and fig. S6) shows a marked dependence. For a 3% doping level, the cloud almost vanishes at temperatures above 10 K, while undoped samples require ≈30 K for an analogous effect.

Kinetic Monte Carlo (KMC) simulations were performed to extract the parameters of electron-polaron migration, see Fig. 3, E to H. The model (detailed description in Materials and Methods) considered electrostatic interactions among all polarons in the slab, including calculation of the interface between the hematite (dielectric permittivity of ε_r_ = 20) (*1*) and vacuum. Polaron hopping used Arrhenius statistics with a frequency prefactor *v*_0_ and activation energy *E*_A_ *+* ∆*E*/2, where ∆*E* is the difference in electrostatic energy between the initial and final states. Figure 3 (E and F) used nearest-neighbor hops in a cubic lattice, while Fig. 3 (G and H) used longer hops without a fixed lattice.

Figure 3 (E to H) shows KMC simulations of the experimental data corresponding to 0.03% Ti doping (Fig. 3B). The commonly assumed model based on nearest neighbor hopping shows severe shortcomings: In Fig. 3E, the hopping distance was set to 0.3 nm, and the prefactor ν_0_ was kept fixed at 10^13^ s^−1^. With these standard parameters, the cloud spreading proceeds in a very narrow temperature range, in contrast to the experimental data that always show a gradual decay over a wide temperature range. This disagreement could, in principle, be corrected by fitting the prefactor ν_0_ as a free parameter in the simulation (see Fig. 3F). Using ν_0_ = 10^5^ s^−1^ provides a good match with the experiment, yet such a low prefactor can be hardly justified (see section 2.3 in the Supplementary Materials).

The experimental data are more consistent with a KMC model based on hopping over longer distances, corresponding to migration via the delocalized state predicted by DFT. In Fig. 3 (D, G, and H), the prefactor was fixed at 10^13^ s^−1^ and the hopping distance was varied. The experimental data can be matched with hopping distances above 2 nm, and the best agreement is achieved for *d* ≈ 25 nm (Fig. 3H). The main difference between the short and long hops is the stronger impact the electric filed (Coulomb repulsion of charge carriers), where the difference of electrostatic energies between the initial and final states is higher. The electric field *E* inside the polaronic clouds is 0.1 to 1 meV/nm (see fig. S7); therefore, the nearest neighbor hops alter the activation barriers by a value of ≈*E* · *d*/2, which is well below 1 meV; this is negligible in comparison to *E*_A_ (see Table 1). Longer hops provide a better agreement with the experimental data; the polaron clouds expand over a wide temperature range due to the synergistic effects of thermal activation and Coulomb repulsion. All activation energies estimated by the KMC simulations are summarized in Table 1, and data for various Ti doping levels are in figs. S6 and S8.

**Table 1.** Activation energies for polaron hopping, extracted from experimental data by KMC simulations. The first four columns show activation energies for electron polaron hopping in nominally undoped and Ti-doped materials. The last column shows results for hole hopping in a Ni-doped sample.

	Undoped	0.03% Ti	0.7% Ti	3% Ti	0.1% Ni (holes)
*d* = 0.3 nm, *v*_0_ = 10^13^ s^−1^	75 meV	54 meV	31 meV	22 meV	230 meV
*d* = 0.3 nm, *v*_0_ = 10^5^ s^−1^ (unphysical)	43 meV	30 meV	15 meV	11 meV	
*d* = 2.5 nm, *v*_0_ = 10^13^ s^−1^	79 meV	58 meV	33 meV	23 meV	
*d* = 25 nm, *v*_0_ = 10^13^ s^−1^	90 meV	67 meV	38 meV	27 meV	

The activation energies estimated by DFT (Fig. 1) are compatible with the KMC analysis of the experimental data for the undoped sample (Table 1). The magnitude is also consistent with electrical transport measurements (*36*–*39*): *n*-doped samples typically show activation energies 0.11 to 0.17 eV, but this value corresponds to a sum of the energy required to escape from the dopant’s Coulomb potential [*E*_i_ ≈ 0.1 eV according to DFT (*31*)] plus the activation energy for polaron hopping *E*_A_. The macroscopic electrical conductivity σ is proportional to the product of the free charge carrier concentration *n*_i_ and the polaron mobility μ (*31*)

σ=ni·μ=nd·e−EikT·ea2nν04kT·e−EakT

where *n*_d_ is the donor concentration, *e* is the electron charge, *a* is the hopping distance, *n* is the number of equivalent neighbor sites, and ν_0_ is the frequency prefactor. In our method, the excess electrons were injected from the tip and do not originate from a dopant; thus, we determine the activation energy for polaron migration alone (20 to 90 meV; see Table 1).

All our experiments indicate isotropic polaron migration, while a strong anisotropy in electrical resistivity would be expected for nearest-neighbor hopping, as often mentioned in the literature (*31*, *40*). The transport data indicate that the anisotropy in conductivity of *n*-doped samples is about one order of magnitude at room temperature, (*36*, *37*), while higher anisotropy was only reported for synthetic undoped crystals (*36*). Samples used in this study showed anisotropy of the electrical resistivity below one order of magnitude (see section 1.2.). The observed isotropic migration supports the picture of electron delocalization, followed by migration over nm distances caused by the electric field.

On the basis of the available experimental results, we set a hypothesis that the presence of extrinsic dopants promotes the transport via delocalization. This is consistent with the reduced anisotropy in electrical conductivity of doped samples (*36*, *41*) and the mobility enhancement in Ti-doped material observed here (Table 1). Already trace Ti doping of 0.1%, i.e., one Ti dopant in a volume of 10 × 10 × 10 Fe atoms, lowers the activation barriers by ~30%. This cannot be explained within nearest-neighbor hopping models because the dopants only influence activation barriers at the nearest neighbor sites (see also DFT analysis in section S3.3) (*31*, *40*), and the lattice sites further away from the dopant remain a bottleneck in macroscopic charge transport. Conversely, the delocalization mechanism appears feasible because the mean Ti-Ti distances are comparable with the hopping distance derived from the KMC analysis (Fig. 2, G and H). From the experimental data, it also appears possible that the transition state could be only partially delocalized, corresponding to a large polaron several nanometers in radius. We note that our DFT calculations could not provide any insight into the mechanism for easier transport in the doped material. The delocalized solution proposed as a transition state could not be modeled by DFT in the presence of Ti, where it did not appear as a metastable solution (in contrast to undoped material, details are discussed in section S3.2).

The experimental data on hole polarons are summarized in Fig. 4, measured on a film doped with 0.1% Ni. Figure 4B shows experiments performed at a base temperature of 4.7 K. Upon annealing up to 20 K, the electrostatic potential measured above the surface did not undergo a substantial decay. Figure 4C shows an experiment performed at a base temperature of 77 K (LN_2_) and with the sample annealed up to 140 K. Initially, there is a slight decay of the electrostatic potential, but the cloud does not evolve further after annealing to higher temperatures. This behavior has been observed several times. It is noteworthy that the hole polaron clouds could not be fully erased by light illumination, which was always possible for electron polaron clouds. The reason is unclear; it may be either related to the low mobility of hole polarons or, possibly, due to the formation of structural defects in the presence of holes.

KMC simulations show that the diffusion of hole polarons cannot be described by a single activation energy. The simulations considered various concentrations of trapping centers, i.e., lattice sites that the polaron cannot escape at the highest experimental temperature of 140 K. A trap concentration of 10^−3^ provides a good match to the experimental data (Fig. 4D); different trap concentrations are shown in fig. S10. The activation energy for a hole polaron estimated from the initial decay of the cloud is 0.23 eV, assuming a pre-factor of 10^13^ s^−1^. This matches the activation energy for hole polaron diffusion estimated by DFT (*29*, *32*).

The concept of hole traps is well established in photocatalysis. One can speculate about the origin of these trapping centers: (i) They can be defects present in the lattice before the polaron injection. Note that the calculated concentration of 10^−3^ matches the level of Ni doping. (ii) The holes can weaken the lattice oxygen bonds and promote defect formation. (iii) The experiments were performed on a p-doped layer grown on a bulk crystal, which is presumably slightly *n*-doped due to oxygen vacancies. The initial decay of the hole cloud could be due to the hole annihilation by underlying electrons, which stops later due to the formation of a *p-n* junction.

In summary, this work used noncontact AFM to image both electron and hole polarons in real space, down to the single quasiparticle limit. AFM appears as a potent tool for nondestructive probing of polaron properties at the atomic scale and principally allows investigating their kinetics induced by electric fields, light, and thermal excitations and tackling details about the transport mechanisms and charge trapping. AFM overcomes the limitations of scanning tunneling microscopy (STM) (*42*–*45*), which relies on the tunneling of millions of electrons per second; this is likely to influence the polaronic configuration and is only applicable to sufficiently conducting systems.

Here, we have identified the delocalization mechanism as an important type of electron polaron transport in hematite. In materials showing shallow small polaron states below the conduction band, polarons can get destabilized by thermal distortions and initiate a free electron–like transport mechanism that competes with the nearest-neighbor hopping. Experimental data indicate that the delocalization of electron polarons can be promoted by Ti dopants or by the presence of high electric fields. This can be relevant, e.g., in *p*-*n* junctions or at solid-liquid interfaces. The methodology introduced here may open a way to understand fundamental mechanisms in polaron physics and thus promote development of better (photo)catalytic materials or single-electron devices.

## MATERIALS AND METHODS

The samples were grown ex-situ by pulsed laser deposition (*21*, *46*); details are described in the Supplementary Materials. The films of Ti-doped hematite were 70- to 100-nm thick, and the doping levels are estimated as (0.035 ± 0.002), (0.77 ± 0.06), and (3.09 ± 0.24) at % Ti. The Ni-doped film was 100-nm thick, and the Ni doping concentration was (0.1 ± 0.01) at %. In the text, all doping concentrations are rounded down.

Ti-doped (*n*-type) samples have been used for studying diffusion of electron polarons, and Ni-doped (*p*-type) samples have been used for hole polarons. This approach has been adopted because the methodology developed here is suitable for studying diffusion of majority carriers. For instance, hopping of holes in *n*-doped samples cannot be studied this way because injecting holes into an *n*-doped surface promotes diffusion of majority (electron) polarons into the hole-injected region at temperatures much lower than required for activation of hole mobility, followed by electron-hole annihilation. Consequently, this configuration would result in measuring the mobility of electron polarons.

Before the AFM measurements, the samples were mounted on tantalum sample plates (Omicron type) and cleaned by cycles of sputtering and annealing to 640°C in a partial oxygen pressure of 5 × 10^−4^ Pa (*47*). A uniform (1 × 1) surface termination was obtained on the Ti-doped samples. The Ni-doped samples typically had residues of the (2 × 1) reconstruction (*20*). Notably, all results related to polaron kinetics appear insensitive to the surface quality and surface termination. This supports the picture of migration through the bulk, rather than on the surface, as deduced also from the KMC simulations.

The combined STM/AFM measurements were performed on commercial cryogenic systems from Scienta Omicron, either the polar or the low temperature STM, in ultrahigh vacuum (UHV). A differential preamplifier (*48*) for the deflection signal was used. Defined tip conditions were obtained by treatment on a Cu (110) surface. A base temperature of 4.7 K was used for most experiments, except for some investigations of hole polaron mobility (see text). LCPD maps were obtained by measuring a grid of Kelvin parabolas and fitting the data. The range of applied biases was strictly controlled to avoid the manipulation of the polarons by the tip electric field: Applying a sample bias too far from the LCPD resulted in modification of the polaron cloud; we typically restricted the bias sweep to ±0.3 V around the LCPD when working with electron polarons (hole polarons were less sensitive to the electric field, allowing more than ±1 V). Controlled sample annealing inside the STM/AFM head was done by a heater placed behind the sample. After ramping the temperature to the setpoint, the sample was kept at the desired temperature for 10 min and cooled back to the base temperature. The tip was retracted by >2 μm during the annealing steps to avoid external electric fields. All experiments were performed in darkness by covering all windows on the UHV chamber, switching off the ion gauge, and keeping the room dark; visible light promotes polaron redistribution. The mechanism of this light-induced charge transport could be either mobilization of a trapped hole itself, or a photon-generated electron-hole pair annihilates a trapped hole and its hole localizes somewhere else.

For bulk-insulator samples, the actual potential drop between a probe tip and the sample is difficult to ascertain because the field penetrates into the insulating sample. We have estimated that approximately 20% of the applied bias is in the junction, and 80% is inside the sample. Several methods were used for this estimation (see the Supplementary Materials). All voltages and LCPD values mentioned in the text are already corrected for this effect, i.e., they are equal to one-fifth of the bias applied on the sample plate.

The collective behavior of the polarons injected into the surface was simulated by a simplified KMC model. The electron and hole polarons were approximated as point charges equivalent to +1 or −1 *e*, respectively. The polarons are allowed to hop to unoccupied sites with the rate *R =* ν_0_ exp[−(*E*_A_ + ∆*E*/2)/*k*_B_*T*], where ν_0_ is the frequency prefactor; *E*_A_ is the diffusion barrier of the isolated (noninteracting) polaron, fitted separately for each hematite doping level; *k*_B_ is the Boltzmann’s constant; and *T* is the temperature. ∆*E* is the difference of the electrostatic interaction energy with the rest of the polarons in the initial and the final positions using the relative permittivity ϵ_r_ = 20. Using ½ ∆*E* in the exponential term assumes that the transition state lies halfway between the initial and final positions. This term might principally lie in the range from 0 *to* ∆*E* depending on the exact hopping mechanism; we have used ½ ∆E in all cases. The interface between the sample and the vacuum was resolved analytically by using mirror charges *q*′*=* (ϵ − 1)/(ϵ + 1)*q*, where *q* is the charge of one polaron (*49*, *50*). The potential above the surface corresponding to the LCPD was calculated using the effective value of the dielectric permittivity ϵ_eff_ = (ϵ_r_ *+* 1)/2 (*49*). Further details are provided in the Supplementary Materials. Two KMC implementations were used: Figs. 3 (E and F) and 4 used a fixed cubic grid of sites with a lattice parameter of 0.3 nm using a rejection-free KMC algorithm. The longer hops in Fig. 3 (G and H) used the Metropolis algorithm without a fixed lattice, where hops of a given length and random direction are generated, and their rejection probability is evaluated on the basis of the electrostatic potential in the initial and final states. Both simulations include the electrostatic interactions between all polarons, leading to a preferential migration in the direction against the gradient of the potential, resulting in spreading of the polaronic cloud.

Our KMC models examined the cases of pure surface diffusion, pure bulk diffusion, and diffusion limited to the Ti-doped layer (see the sample geometry in Fig. 2A; the Ti-doped layer has a higher electron mobility than the underlying undoped sample). A good match with the experimental data was obtained for diffusion restricted to the doped layer or for pure bulk diffusion. Pure surface diffusion seems unlikely (see fig. S9).

Spin-resolved DFT calculations were performed using the Vienna ab initio simulation package (*51*, *52*). We adopted the HSE06 hybrid functional (*53*) with 12% of Hartree-Fock mixing. This setup, recently proposed by Ahart (*29*), reproduces the experimental bandgap in hematite (*29*, *30*) and provides activation energies for hopping that are lower than in previous theoretical works (*30*, *40*, *54*, *55*) but seem to match the experiments better. We note that the commonly accepted mixing value of 25% leads to an inaccurate description of the electronic properties of hematite: The bandgap is notably overestimated (3.5 eV in contrast to the experimental observations of approximately 2 eV); the electron polaron qualitatively maintains the charge distribution as in the calculations with 12% of mixing (i.e., the charge is equivalently shared on two Fe sites), but the polaronic state is found at deeper energies (more than 1 eV below the conduction band, instead of 0.4 eV) and the polaron formation energy is unphyiscally high (0.5 eV instead of 56 meV); moreover, the mixing of 25% has been reported to violate Koopman conditions (*29*, *56*).

A plane-wave energy cutoff of 550 eV was used and the Γ point for sampling the reciprocal space. The hematite computational cell was a 4 × 4 × 1 super cell (with 480 atoms). This computational setup allows us to obtain an accurate description of the polaron properties in hematite, as systematically analyzed in the literature (*30*, *57*). In cells that are not neutral, the excess hole/electron required to form a polaron was introduced by modifying the total number of electrons and adding a compensating background charge to keep overall charge neutrality. All the atomic coordinates were relaxed to obtain residual forces smaller than 0.01 eV/Å. The delocalized solution was modeled by initially keeping atomic coordinates fixed while populating the bottom of the conduction band of pristine hematite with one excess electron and, lastly, relaxing all coordinates. The charge-density isosurfaces were calculated for the in-gap polaronic peaks appearing in the density of states. The degree of localization of polarons was evaluated by a projection of the polaronic state into the local atomic orbitals (the corresponding local magnetic moments match the values from previous analysis) (*29*). The polaron hopping was modeled by linearly interpolating the atomic coordinates between the initial and the final structures (*58*): The saddle points in Fig. 2 (C to E) represent the energy barriers of the corresponding electron-transfer process.

## Supplementary Materials

### The PDF file includes:

Sections S1 to S4 Figs. S1 to S14 Tables S1 to S4 Legend for movie S1 Legends for data S1 and S2 References

## References

[1] G. S. Parkinson, Iron oxide surfaces. Surf. Sci. Rep. 71, 272–365 (2016).

[2] K. Sivula, F. L. Formal, M. Grätzel, Solar water splitting: Progress using hematite (α-Fe_2_O_3_) photoelectrodes. Chem. Sus. Chem. 4, 432–449 (2011). 10.1002/cssc.201000416 21416621

[3] J. Zhang, S. Eslava, Understanding charge transfer, defects and surface states at hematite photoanodes. Sust. Energy Fuels 3, 1351–1364 (2019).

[4] C. Franchini, M. Reticcioli, M. Setvin, U. Diebold, Polarons in materials. Nat. Rev. Mater. 6, 560–586 (2021).

[5] I. G. Austin, N. F. Mott, Polarons in crystalline and non-crystalline materials. Adv. Phys. 50, 757–812 (2001).

[6] V. Coropceanu, J. Cornil, D. A. S. Filho, Y. Olivier, R. Silbey, J.-L. Brédas, Charge transport in organic semiconductors. Chem. Rev. 107, 926–952 (2007). 17378615 10.1021/cr050140x

[7] F. Ortmann, F. Bechstedt, K. Hannewald, Charge transport in organic crystals: Theory and modelling. Phys. Stat. Solidi B 248, 511–525 (2011).

[8] Y. Natanzon, A. Azulay, Y. Amouyal, Evaluation of polaron transport in solids from first-principles. Israel J. Chem. 60, 768–786 (2020).

[9] J. L. M. van Mechelen, D. van der Marel, C. Grimaldi, A. B. Kuzmenko, N. P. Armitage, N. Reyren, H. Hagemann, I. I. Mazin, Electron-phonon interaction and charge carrier mass enhancement in SrTiO_3_. Phys. Rev. Lett. 100, 226403 (2008). 18643435 10.1103/PhysRevLett.100.226403

[10] C. D. Valentin, G. Pacchioni, A. Selloni, Reduced and n-Type doped TiO_2_: Nature of Ti^3+^ Species. J. Phys. Chem. C 113, 20543–20552 (2009).

[11] A. C. Papageorgiou, N. S. Beglitis, C. L. Pang, G. Teobaldi, G. Cabailh, Q. Chen, A. J. Fisher, W. A. Hofer, G. Thornton, Electron traps and their effect on the surface chemistry of TiO_2_(110). Proc. Natl. Acad. Sci. U.S.A. 107, 2391–2396 (2010). 20133773 10.1073/pnas.0911349107 PMC2823884

[12] J. M. D. Teresa, M. R. Ibarra, P. A. Algarabel, C. Ritter, C. Marquina, J. Blasco, J. García, A. Moral, Z. Arnold, Evidence for magnetic polarons in the magnetoresistive perovskites. Nature 386, 256–259 (1997).

[13] C. Jooss, L. Wu, T. Beetz, R. F. Klie, M. Beleggia, M. A. Schofield, S. Schramm, J. Hoffmann, Y. Zhu, Polaron melting and ordering as key mechanisms for colossal resistance effects in manganites. Proc. Natl. Acad. Sci. U.S.A. 104, 13597–13602 (2007). 17699633 10.1073/pnas.0702748104 PMC1959427

[14] S. Song, J. Kim, D. Lee, J. Lee, T. Min, J.-A. Chae, J.-S. Bae, J. Lee, J.-S. Lee, S. Park, The effect of Fe^2+^ state in electrical property variations of Sn-doped hematite powders. J. Am. Ceram. Soc. 100, 3928–3934 (2017).

[15] D. Cogan, L. A. Lonegran, Electrical conduction in Fe_2_O_3_ and Cr_2_O_3_. Sol. State Commun. 15, 1517–1519 (1974).

[16] T. J. Smart, A. C. Cardiel, F. Wu, K.-S. Choi, Y. Ping, Mechanistic insights of enhanced spin polaron conduction in CuO through atomic doping. NPJ Computat. Mater. 4, 61 (2018).

[17] R. Karsthof, M. Grundmann, A. M. Anton, F. Kremer, Polaronic interacceptor hopping transport in intrinsically doped nickel oxide. Phys. Rev. B 99, 235201 (2019).

[18] W. Zhang, F. Wu, J. Li, D. Yan, J. Tao, Y. Ping, M. Liu, Unconventional relation between charge transport and photocurrent via boosting small polaron hopping for photoelectrochemical water splitting. ACS Energy Lett. 3, 2232–2239 (2018).

[19] M. A. Henderson, Insights into the (1 × 1) to (2 × 1) phase transition of the α-Fe_2_O_3_(012) surface using EELS LEED and water TPD. Surf. Sci. 515, 253–262 (2002).

[20] F. Kraushofer, Z. Jakub, M. Bichler, J. Hulva, P. Drmota, M. Weinold, M. Schmid, M. Setvin, U. Diebold, P. Blaha, G. S. Parkinson, Atomic-scale structure of the hematite α-Fe_2_O_3_(1-102) “R-Cut” surface. J. Phys. Chem. C 122, 1657–1669 (2018). 10.1021/acs.jpcc.7b10515 PMC5823487 29492182

[21] G. Franceschi, F. Kraushofer, M. Meier, G. S. Parkinson, M. Schmid, U. Diebold, M. Riva, A model system for photocatalysis: Ti-doped α-Fe_2_O_3_(11̅02) single-crystalline films. Chem. Mater. 32, 3753–3764 (2020). 32421058 10.1021/acs.chemmater.9b04908 PMC7222102

[22] A. G. Tamirat, J. Rick, A. A. Dubale, W.-N. Su, B.-J. Hwang, Using hematite for photoelectrochemical water splitting: A review of current progress and challenges. Nanoscale Horiz. 1, 243–267 (2016). 32260645 10.1039/c5nh00098j

[23] C. Li, Z. Luo, T. Wang, J. Gong, Surface, bulk, and interface: Rational design of hematite architecture toward efficient photo-electrochemical water splitting. Adv. Mater. 30, e1707502 (2018). 29750372 10.1002/adma.201707502

[24] F. J. Giessibl, The qPlus sensor, a powerful core for the atomic force microscope. Rev. Sci. Instrum. 90, 011101 (2019). 30709191 10.1063/1.5052264

[25] S. Sadewasser, T. Glatzel, *Kelvin Probe Force Microscopy* (Springer-Verlag, 2012).

[26] L. Gross, F. Mohn, P. Liljeroth, J. Repp, F. J. Giessibl, G. Meyer, Measuring the charge state of an adatom with noncontact atomic force microscopy. Science 324, 1428–1431 (2009). 19520956 10.1126/science.1172273

[27] M. Setvin, J. Hulva, G. S. Parkinson, M. Schmid, U. Diebold, Electron transfer between anatase TiO_2_ and an O_2_ molecule directly observed by atomic force microscopy. Proc. Natl. Acad. Sci. U.S.A. 114, E2556–E2562 (2017). 28289217 10.1073/pnas.1618723114 PMC5380104

[28] S. Fatayer, B. Schuler, W. Steurer, I. Scivetti, J. Repp, L. Gross, M. Persson, G. Meyer, Reorganization energy upon charging a single molecule on an insulator measured by atomic force microscopy. Nat. Nanotechnol. 13, 376–380 (2018). 29662243 10.1038/s41565-018-0087-1

[29] C. S. Ahart, K. M. Rosso, J. Blumberger, Electron and hole mobilities in bulk hematite from spin-constrained density functional theory. J. Am. Chem. Soc. 144, 4623–4632 (2022). 35239359 10.1021/jacs.1c13507 PMC9097473

[30] N. Iordanova, M. Dupuis, K. M. Rosso, Charge transport in metal oxides: A theoretical study of hematite α-Fe_2_O_3_. J. Chem. Phys. 122, 144305 (2005). 15847520 10.1063/1.1869492

[31] M. Chen, A. C. Grieder, T. J. Smart, K. Mayford, S. McNair, A. Pinongcos, S. Eisenberg, F. Bridges, Y. Li, Y. Ping, The impacts of dopants on the small polaron mobility and conductivity in hematite–the role of disorder. Nanoscale 15, 1619–1628 (2023). 36602002 10.1039/d2nr04807h

[32] C. Cheng, Y. Zhu, Z. Zhou, R. Long, W.-H. Fang, Photoinduced small electron polarons generation and recombination in hematite. NPJ Computat. Mater. 8, 148 (2022).

[33] A. V. Barzykin, M. Tachiya, Mechanism of charge recombination in dye-sensitized nanocrystalline semiconductors: random flight model. J. Phys. Chem. B 106, 4356–4363 (2002).

[34] J. Nelson, S. A. Haque, D. R. Klug, J. R. Durrant, Trap-limited recombination in dye-sensitized nanocrystalline metal oxide electrodes. Phys. Rev. B 63, 205321 (2001).

[35] F. Mortreuil, L. Boudou, K. Makasheva, G. Teyssedre, C. Villeneuve-Faure, Influence of dielectric layer thickness on charge injection, accumulation and transport phenomena in thin silicon oxynitride layers: A nanoscale study. Nanotechnology 32, 065706 (2021). 33086199 10.1088/1361-6528/abc38a

[36] D. Benjelloun, J. P. Bonnet, J. P. Doumerc, J. C. Launey, M. Onillon, Anisotropy in the electrical properties of zirconium-doped a-Fe_2_O_3_ single crystals. Mat. Chem. Phys. 20, 1–12 (1988).

[37] T. Nakau, Electrical conductivity of a-Fe_2_O_3_. J. Phys. Soc. Jap. 15, 727 (1960).

[38] C. M. Tian, W.-W. Li, Y. M. Lin, Z. Z. Yang, L. Wang, Y. G. Du, H. Y. Xiao, L. Qiao, J. Y. Zhang, L. Chen, D.-C. Qi, J. L. MacManus-Driscoll, K. H. L. Zhang, Electronic structure, optical properties, and photoelectrochemical activity of Sn-doped Fe_2_O_3_ thin films. J. Phys. Chem. C 124, 12548–12558 (2020).

[39] C. Sanchez, K. Sieber, G. Somorjai, The photoelectrochemistry of niobium doped α-Fe_2_O_3_. J. Electroanal. Chem. Interfacial Electrochem. 252, 269–290 (1988).

[40] Z. Zhou, R. Long, O. V. Prezhdo, Why silicon doping accelerates electron polaron diffusion in hematite. J. Am. Chem. Soc. 141, 20222–20233 (2019). 31791126 10.1021/jacs.9b10109

[41] D. Benjelloun, J.-P. Bonnet, P. Dordor, J.-C. Launay, M. Onillon, P. Hagenmuller, Anisotropie des propriétés électriques de monocristaux de Fe_2_0_3_ dopés au nickel. Rev. de Chemie Minerale 21, 781 (1984).

[42] H. Liu, A. Wang, P. Zhang, C. Ma, C. Chen, Z. Liu, Y.-Q. Zhang, B. Feng, P. Cheng, J. Zhao, L. Chen, K. Wu, Atomic-scale manipulation of single-polaron in a two-dimensional semiconductor. Nat. Commun. 14, 3690 (2023). 37344475 10.1038/s41467-023-39361-0 PMC10284845

[43] M. Cai, M.-P. Miao, Y. Liang, Z. Jiang, Z.-Y. Liu, W.-H. Zhang, X. Liao, L.-F. Zhu, D. West, S. Zhang, Y.-S. Fu, Manipulating single excess electrons in monolayer transition metal dihalide. Nat. Commun. 14, 3691 (2023). 37344472 10.1038/s41467-023-39360-1 PMC10284909

[44] C. M. Yim, M. B. Watkins, M. J. Wolf, C. L. Pang, K. Hermansson, G. Thornton, Engineering polarons at a metal oxide surface. Phys. Rev. Lett. 117, 116402 (2016). 27661706 10.1103/PhysRevLett.117.116402

[45] M. Setvin, C. Franchini, X. Hao, M. Schmid, A. Janotti, M. Kaltak, C. G. V. d. Walle, G. Kresse, U. Diebold, A direct view at excess electrons in TiO_2_ rutile and anatase. Phys. Rev. Lett. 113, 086402 (2014). 25192111 10.1103/PhysRevLett.113.086402

[46] S. Gerhold, M. Riva, B. Yildiz, M. Schmid, U. Diebold, Adjusting island density and morphology of the SrTiO_3_(110)-(4 × 1) surface: Pulsed laser deposition combined with scanning tunneling microscopy. Surf. Sci. 651, 76–83 (2016).

[47] G. Franceschi, M. Schmid, U. Diebold, M. Riva, Reconstruction changes drive surface diffusion and determine the flatness of oxide surfaces. J. Vac. Sci. Technol. A 40, 023206 (2022).

[48] F. Huber, F. J. Giessibl, Low noise current preamplifier for qPlus sensor deflection signal detection in atomic force microscopy at room and low temperatures. Rev. Sci. Instrum. 88, 073702 (2017). 28764492 10.1063/1.4993737

[49] W. Greiner, *Classical Electrodynamics* (Springer, 1998).

[50] B. Nadler, U. Hollerbach, R. S. Eisenberg, Dielectric boundary force and its crucial role in gramicidin. Phys. Rev. E 68, 021905 (2003). 10.1103/PhysRevE.68.021905 14525004

[51] G. Kresse, J. Furthmuller, Efficiency of ab-initio total energy calculations for metals and semiconductors using a plane-wave basis set. Comput. Mater. Sci. 6, 15–50 (1996).

[52] G. Kresse, D. Joubert, From ultrasoft pseudopotentials to the projector augmented-wave method. Phys. Rev. E 59, 1758–1775 (1999).

[53] A. V. Krukau, O. A. Vydrov, A. F. Izmaylov, G. E. Scuseria, Influence of the exchange screening parameter on the performance of screened hybrid functionals. J. Chem. Phys. 125, 224106 (2006). 17176133 10.1063/1.2404663

[54] N. Adelstein, J. B. Neaton, M. Asta, L. C. D. Jonghe, Density functional theory based calculation of small-polaron mobility in hematite. Phys. Rev. B 89, 245115 (2014).

[55] T. J. Smart, Y. Ping, Effect of defects on the small polaron formation and transport properties of hematite from first-principles calculations. J. Phys. Cond. Matt. 29, 394006 (2017). 10.1088/1361-648X/aa7e3d 28685710

[56] C. S. Ahart, J. Blumberger, K. M. Rosso, Polaronic structure of excess electrons and holes for a series of bulk iron oxides. Phys. Chem. Chem. Phys. 22, 10699–10709 (2020). 32091520 10.1039/c9cp06482f

[57] N. Ansari, K. Ulman, M. F. Camellone, N. Seriani, R. Gebauer, S. Piccinin, Hole localization in Fe_2_O_3_ from density functional theory and wave-function-based methods. Phys. Rev. Mater. 1, 035404 (2017).

[58] N. Deskins, M. Dupuis, Electron transport via polaron hopping in bulk TiO2: A density functional theory characterization. Phys. Rev. E 75, 195212 (2007).

[59] G. Franceschi, F. Kraushofer, M. Meier, G. S. Parkinson, M. Schmid, U. Diebold, M. Riva, A model system for photocatalysis: Ti-Doped α-Fe_2_O_3_(1102) single-crystalline films. Chem. Mater. 32, 3753–3764 (2020). 32421058 10.1021/acs.chemmater.9b04908 PMC7222102

[60] A. S. Lucier, H. Mortesen, Y. Sun, P. Grutter, Determination of the atomic structure of scanning probe microscopy tungsten tips by field ion microscopy. Phys. Rev. B 72, 235420 (2005).

[61] M. Setvin, J. Javorsky, D. Turcinkova, I. Matolinova, P. Sobotik, P. Kocan, I. Ostadal, Ultrasharp tungsten tips—Characterization and nondestructive cleaning. Ultramicroscopy 113, 152–157 (2012).

[62] M. N. O. Sadiku, R. C. Garcia, Monte Carlo floating random walk solution of Poisson’s equation, in *Southeastcon 1993 Proceedings* (IEEE, 1993).

[63] A. Kuznetsov, A. Sipin, Monte Carlo algorithms for the extracting of electrical capacitance. Mathematics 9, 2922 (2021).

[64] J. C. Papaioannou, G. S. Patermarakis, H. S. Karayianni, Electron hopping mechanism in hematite (α-Fe_2_O_3_). J. Phys. Chem. Sol. 66, 839–844 (2005).

[65] M. Reticcioli, Z. Wang, M. Schmid, D. Wrana, L. A. Boatner, U. Diebold, M. Setvin, C. Franchini, Competing electronic states emerging on polar surfaces. Nat. Commun. 13, 4311 (2022). 35879300 10.1038/s41467-022-31953-6 PMC9314351

[66] S. Giannini, J. Blumberger, Charge transport in organic semiconductors: The perspective from nonadiabatic molecular dynamics. Acc. Chem. Res. 55, 819–830 (2022). 35196456 10.1021/acs.accounts.1c00675 PMC8928466

[67] S. Onari, T. Arai, K. Kudo, Infrared lattice vibrations and dielectric dispersion in α−Fe_2_O_3_. Phys. Rev. B 16, 1717–1721 (1977).

[68] R. A. Lunt, A. J. Jackson, A. Walsh, Dielectric response of Fe_2_O_3_ crystals and thin films. Chem. Phys. Lett. 586, 67–69 (2013).

[69] L. J. Lauhon, W. Ho, Direct observation of the quantum tunneling of single hydrogen atoms with a scanning tunneling microscope. Phys. Rev. Lett. 85, 4566–4569 (2000). 11082597 10.1103/PhysRevLett.85.4566

[70] C. T. Campbell, R. V. Sellers, Enthalpies and entropies of adsorption on well-defined oxide surfaces: experimental measurements. Chem. Rev. 113, 4106–4135 (2013). 23441680 10.1021/cr300329s

[71] E. D. Grave, L. H. Bowen, D. D. Amarasiriwardena, R. E. Vandenberghe, ^57^Fe Mosbauer effect study of highly substituted aluminum hematites: Determination of the magnetic hyperfine field distributions. J. Magnet. Magnetic Mater. 72, 129–140 (1988).

[72] P. Sobotik, P. Kocan, I. Ostadal, Direct observation of Ag intercell hopping on the Si(111)-(7 x 7) surface. Surf. Sci. 537, L442–L446 (2003).

[73] S. V. Divinski, A. Pokoev, N. Esakkiraja, A. Paul, A mystery of “sluggish diffusion” in high-entropy alloys: the truth or a myth? ArXiv: 1804.03465 [cond-mat.mtrl-sci] (2018).

[74] M. Kluge, H. R. Schober, Diffusion and jump-length distribution in liquid and amorphous Cu33Zr67. Phys. Rev. B 70, 224209 (2004).

