# Supplementary material for: Real-space investigation of polarons in hematite Fe2O3
Source: Sci Adv. 2024 Nov 1;10(44):eadp7833. doi: 10.1126/sciadv.adp7833 (PMC11529705; doi:10.1126/sciadv.adp7833)
Supplement: Supplementary file 1 — Sections S1 to S4 Figs. S1 to S14 Tables S1 to S4 Legend for movie S1 Legends for data S1 and S2 References [file sciadv.adp7833_sm.pdf]

Science Advances
AAAS

# Supplementary Materials for

## Real-space investigation of polarons in hematite Fe2O3

Jesus Redondo *et al.*

Corresponding author: Martin Setvin, martin.setvin@mff.cuni.cz; Cesare Franchini, cesare.franchini@univie.ac.at

*Sci. Adv.* **10**, eadp7833 (2024)
DOI: 10.1126/sciadv.adp7833

**The PDF file includes:**

Sections S1 to S4
Figs. S1 to S14
Tables S1 to S4
Legend for movie S1
Legends for data S1 and S2
References

**Other Supplementary Material for this manuscript includes the following:**

Movie S1
Data S1 and S2

# 1. Supplemental Experimental Information

## 1.1. Film growth by pulsed laser deposition

The samples were grown ex-situ by pulsed laser deposition (*59*). Natural $\alpha$-$Fe_2O_3$(1−102) single crystals (SurfaceNet GmbH, one-side polished) were used as substrates for film growth. Prior to the growth, the substrates were cleaned by sonication in neutral detergent (Extran MA02) and ultrapure water, and by cycles of sputtering–annealing (800–1000 °C, 0.2–1 mbar $O_2$) in the growth chamber (*46*), until no naturally occurring contamination was visible in X-ray photoelectron spectroscopy and low-energy electron diffraction. Growth of the doped films was performed by alternating deposition from two targets, as described in detail in Ref. 1. Home-made Ti- and Ni-doped $Fe_2O_3$ targets were used for the 0.03 at.% Ti- and 0.1 at.% Ni-doped films, respectively. The targets were prepared by mixing appropriate amounts of high-purity (> 99.995%) $Fe_2O_3$ and dopant ($TiO_2$ and NiO, respectively) powders in an $HNO_3$-cleaned agate mortar. The powder was then isostatically pressed (400 MPa, 5 min) in freshly made silicone

molds and sintered — embedded in buffer $Fe_2O_3$ powder — inside a Pt crucible (1 bar $O_2$, 1200 °C, 6 h). A single-crystalline $TiO_2$ target was used for films with higher Ti doping levels (0.7 at.% and 3 at.%). For all films, the pure-iron source was a single-crystalline $Fe_3O_4$ target. Table ST1 summarizes the growth conditions used for each film. The growth temperatures were chosen to ensure sufficient diffusion of the dopants to achieve a uniform mixing despite the alternation between targets (and low enough to prevent loss to the bulk or major segregation to the surface). Doping levels were estimated as in Ref. 1 (0.7 at.% Ti and 3 at.% Ti) or by measuring the growth rates from each target by reflection high-energy electron diffraction in the same setup at the same conditions used for film growth (0.03 at.% Ti, 0.1 at.% Ni). The 3 at.% Ti-doped film was approximately 70 nm thick, the 0.77 at.% film was 90 nm thick; the other films were approximately 100 nm thick.

***Table ST1. Pulsed-laser deposition conditions used for the growth of films used in this manuscript.*** *Common to all films: Laser fluence (2 J cm$^{-2}$), repetition frequency (5 Hz), $O_2$ background pressure (2 Pa). "No. pulses" refers to how many times the laser was shot on each target before switching to the other. For each film, the targets were switched "No. repeats" times. For full details see Ref. 1.*

| Doping level | *No. pulses $Fe_3O_4$* | No. pulses secondary | No. repeats | Secondary | *T (°C)* | Thicknes s (nm) |
|---|---|---|---|---|---|---|
| 0.035 at.% Ti | 300 | 9 | 196 | 1% Ti:$Fe_2O_3$ | 850 | 100 |
| 0.77 at.% Ti | 500 | 1 | 120 | $TiO_2$ | 850 | 90 |
| 3.1 at.% Ti | 375 | 3 | 120 | $TiO_2$ | 850 | 70 |
| 0.1 at.% Ni | 382 | 6 | 154 | 5% Ni:$Fe_2O_3$ | 780 | 100 |

## 1.2. Electrical resistivity of the material

The electrical resistivity and anisotropy were measured at room temperature on the sample doped by 0.035% Ti. The conductivity measurements were first performed on the thin film, then the film was mechanically polished off and finally the nominally undoped substrate was measured. The resistivity of the film was calculated considering the film thickness and the value of the substrate resistivity. Van der Pauw configuration of electrodes was used, the sample edges were parallel with the [1-10-1] and [11-20] directions. The [11-20] direction lies within the plane of Fe atoms with the same spin. The [1-10-1] direction is not exactly perpendicular to this plane, but this should have a negligible effect on the estimation of the anisotropy.

For the nominally undoped (natural) material, the average resistivity was $9.5 \times 10^8$ Ω cm. The resistivity in [11-20] direction was 5× lower than in the [1-10-1] direction.

For the film doped with 0.035% Ti, the average resistivity was $4.8 \times 10^5$ Ω cm and the resistivity in the [11-20] direction was 6× lower than in the [1-10-1] direction.

## 1.3. Surface structure and morphology

The polaron injection experiments were carried out in thin (≈100 nm) Ti and Ni doped $\alpha-F_2O_3(1{-}102)$ films grown on natural $\alpha-F_2O_3(1{-}102)$ crystals. This plane of hematite can accommodate two surface terminations in ultra-high vacuum: a bulk-truncated (1 × 1) phase and a (2 × 1) reduced

reconstruction (*20*). All samples were prepared by sputtering in $Ar^+$ and subsequent annealing in $10^{-4}$ Pa $O_2$ up to 620 °C, which yields the stochiometric (1 × 1) termination in undoped hematite crystals. A flat surface morphology with large terraces was obtained by a procedure described in Ref. (*47*). Extensive sputtering was avoided due to the limited thickness of the doped films. For the undoped hematite substrates used for layer growth, low-energy electron diffraction (LEED) patterns could not be acquired at room temperature due to charging, indicating a low doping level.

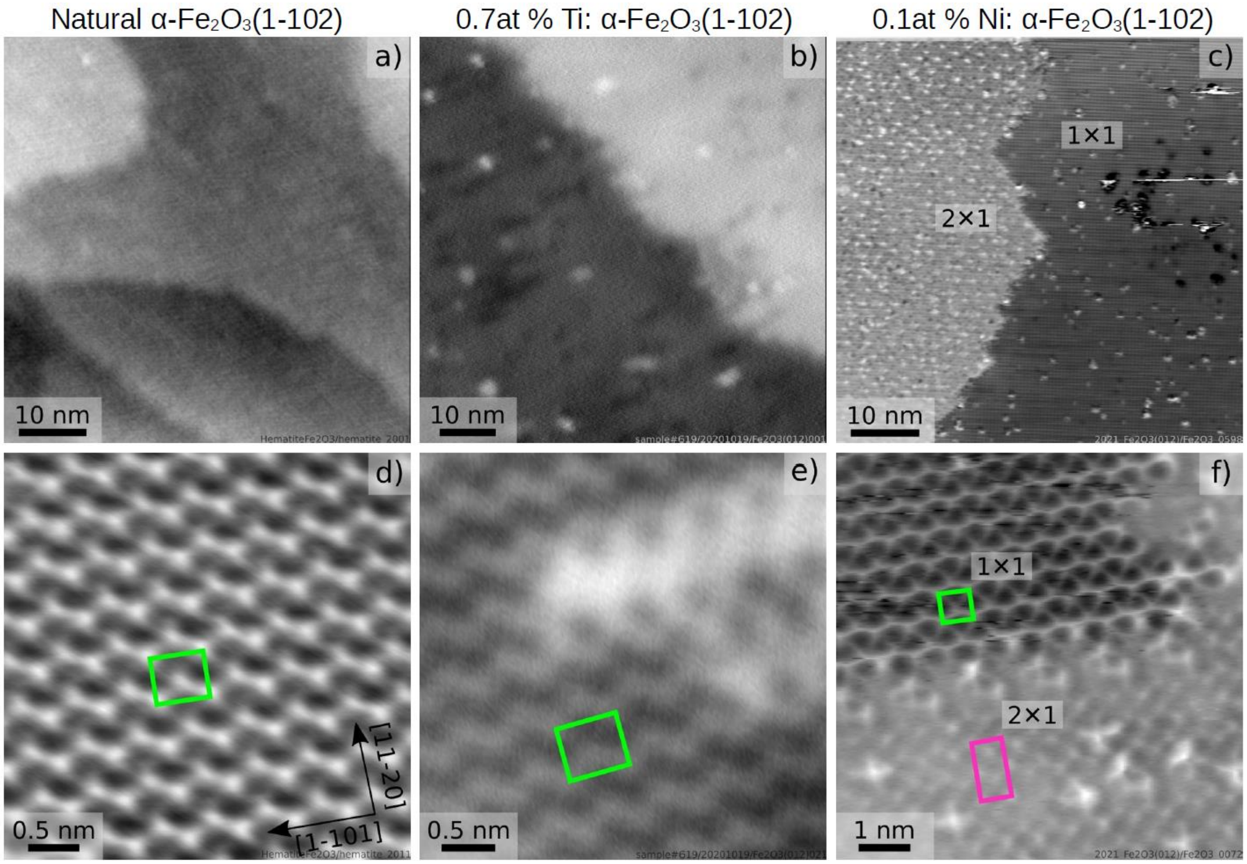


***Figure S1. The surfaces of natural, Ti and Ni-doped α-$F_2O_3$(1–102), imaged with nc-AFM.*** *The top row (panels a,b,c) shows an overview of the surface morphology, the bottom row (panels d,e,f) shows small-area images with atomic resolution. a) The surface of a natural, nominally undoped sample, 60 × 60 $nm^2$. b) 0.7% Ti-doped sample, 50 × 50 $nm^2$. c) 0.1% Ni-doped surface (60 × 60 $nm^2$), showing a mixture of (2×1) and (1 × 1) domains (top and bottom part of panel c). All images measured at T = 4.7 K. Green squares mark the 1 × 1 unit cell.*

Figure S1 shows representative nc-AFM images of the samples used. The top row shows overview images. For the natural (nominally undoped) samples, the surface is homogeneously covered by the (1 × 1) bulk termination. The Ni-doped crystals show a mixture of (1 × 1) and (2 × 1) phases, which is presumably associated with the Ni dopants and a different Fermi level position. The Ni doped samples required annealing in a higher partial pressure of oxygen (order of $10^{-5}$ mbar) to form a predominantly (1 × 1) terminated surface.

The bottom row of panels in Fig. S1 shows atomically resolved constant-height AFM images for the different types of doping. Undoped surfaces are largely defect-free (Fig. S1a,d). The Ti-doped surfaces

show trenches that are associated with Ti dopants; details of Ti-doped surfaces were discussed elsewhere (*59*). The Ni-doped surfaces show point defects that are tentatively attributed to surface Ni dopants.

It is noteworthy that the polaron-related experiments presented in the main text did not show any significant dependence on the exact surface condition or reconstruction. In other words, the presence of surface impurities or a different surface reconstruction did not substantially alter the migration of polarons. This is consistent with the conclusion that the polaron migration predominantly proceeds in the bulk in all the investigated samples.

### 1.4. Effective bias in the tunneling junction

All measurements were performed on bulk single crystals with a thickness of ≈2 mm. In such a geometry, applying the sample bias into the tunneling junction is not straightforward: A voltage (nominal bias potential) is applied between the supporting sample plate (tantalum) and the tip. The quantity relevant for the experiment is the potential drop between the tip and the sample surface, however. This is akin to a dielectric material partly filling the gap of a capacitor: The dielectric changes the field distribution. The total potential difference between the two electrodes falls off more gradually within the dielectric than in vacuum and the potential drop in the gap between the bottom and the upper electrode (here the tip) is changed. This quantity is estimated in the following and referred to as 'effective bias'.

The effective bias determines the tunneling conditions and (together with the work function difference between the tip and the surface) the electrostatic forces. It depends on the geometry, *e.g.*, on the tip radius and the tip-sample distance, and inside the dielectric the electrostatic potential is not the same for all surface and subsurface atoms. We use an approximation where the effective bias is evaluated as a fraction of the nominal bias applied to the sample plate. We have used several methods for estimating the effective bias in the tunneling junction and came to a consistent result that ≈20% of the applied potential is in the tunneling junction and 80% is in the bulk sample.

All bias values quoted in the main text and the supplement are the effective values, *i.e.*, one fifth of the applied bias. It means that injecting electron polarons typically required applying a bias of +4 to +5 V on the sample plate, while we quote an effective bias of +0.8 to +1.0 V. Injecting hole polarons was typically performed with a bias of -5 to -6 V, corresponding to effective values of -1.0 to -1.2 V. The use of effective bias values allows comparing the voltages to the band structure of the material and to the electrostatic potentials computed in the KMC simulations.

As the first step, we have estimated the effective bias from the Poisson equation, using both simplified analytical solutions and full numerical calculations of a realistic tip shape. The experimental configuration resembles the geometry in field emission experiments, therefore we profit from experience gained in this area (*60, 61*). As the first approximation of the layout of the electrostatic potential, we have used the concept of mirror charges (*50*), see Fig. S2a. When a point charge is placed near an interface between two dielectrics (here vacuum and the sample with $\varepsilon_r$ = 20), the equipotential surfaces between the charge and the interface can be approximated by a spherical shape. This configuration can therefore be used as a simple analytical model of a spherical tip at a specific potential, kept at a certain distance from the sample. In Fig. S2a, a charge of +170 *e* was placed 20 nm from the interface. The electrostatic potential along the z axis is plotted in Fig. 1b (blue line). Approximately 1/3 of the applied potential drops

within the sample, while 2/3 of the potential drops within the tunneling junction (within one nanometer from the sample). For comparison, a hypothetical sample with a permittivity $\varepsilon_r = 1$ is shown in Fig. S2a; here only 5% of the potential drop would be in the tunneling junction.

Approximating the tip by a sphere is an oversimplification. From field emission in vacuum it is known that realistic tip shapes reduce the electric field near the tip by the so called field reduction factor (*60*), which ranges from 3 to 8 for most of the tips, but can be as high as 100 (*61*). An exact quantification of the electrostatic potential requires a full numerical simulation based on a realistic layout of electrodes. We have used the Monte Carlo floating random walk algorithm (*62*) modified for modelling the dielectric permittivity of the sample (*63*). The algorithm was first tested on analytical cases, see for example open squares in Fig. S2b. Here we have modeled a sphere with a radius of 20 nm, placed 1 nm from the sample.

A model used from mimicking the realistic experimental configurations is shown in Fig. S2c. The tip is modelled as a sphere with a diameter of r, attached to a conical shaft with an opening angle of α. The tip was held at a potential of 1, at the same potential as the boundary condition behind it. The back side of the sample (as well as the boundaries, which were at sufficiently large distances) was set to a potential of zero. A sample thickness $d_{\text{sample}}$ = 2 mm was used; the distances to the boundaries were $d_{\text{back}}$ = 3 mm and $d_{\text{side}}$ = 5 mm. Geometries assuming tips with radii ranging from 20 to 200 nm and a cone angle α ranging from 5 to 90 degrees were considered realistic. In these configurations, 20 to 50% of the applied potential drops between the tip and the sample. Some examples are shown in Fig. S2d; a detailed study on this topic will be published elsewhere.

In this work, we have consistently used the lower value of 20% because tips used in q-plus AFM are typically not very sharp. The estimated rescaling factor of 20% is consistent with the comparison of the calculated potentials derived from the number of injected charge carriers and the experimental values. Also, the voltage difference between injection of electrons and holes is comparable to the band gap of hematite.

Last, the LCPDs measured on the hematite samples directly after approaching the tip provides certain information on the rescaling factor. The LCPD on hematite was typically in the range from +0.5 to +2.0 V (for Ti-doped samples, the real biases voltages without rescaling are quoted here). All tips were prepared and calibrated by treatment on the Cu (110) surface, where the LCPD was ranging from -0.1 to +0.2 V. Thus, comparing the spread of the real different tip work functions with the measured LCPD on hematite is again consistent with the rescaling factor of 20%.

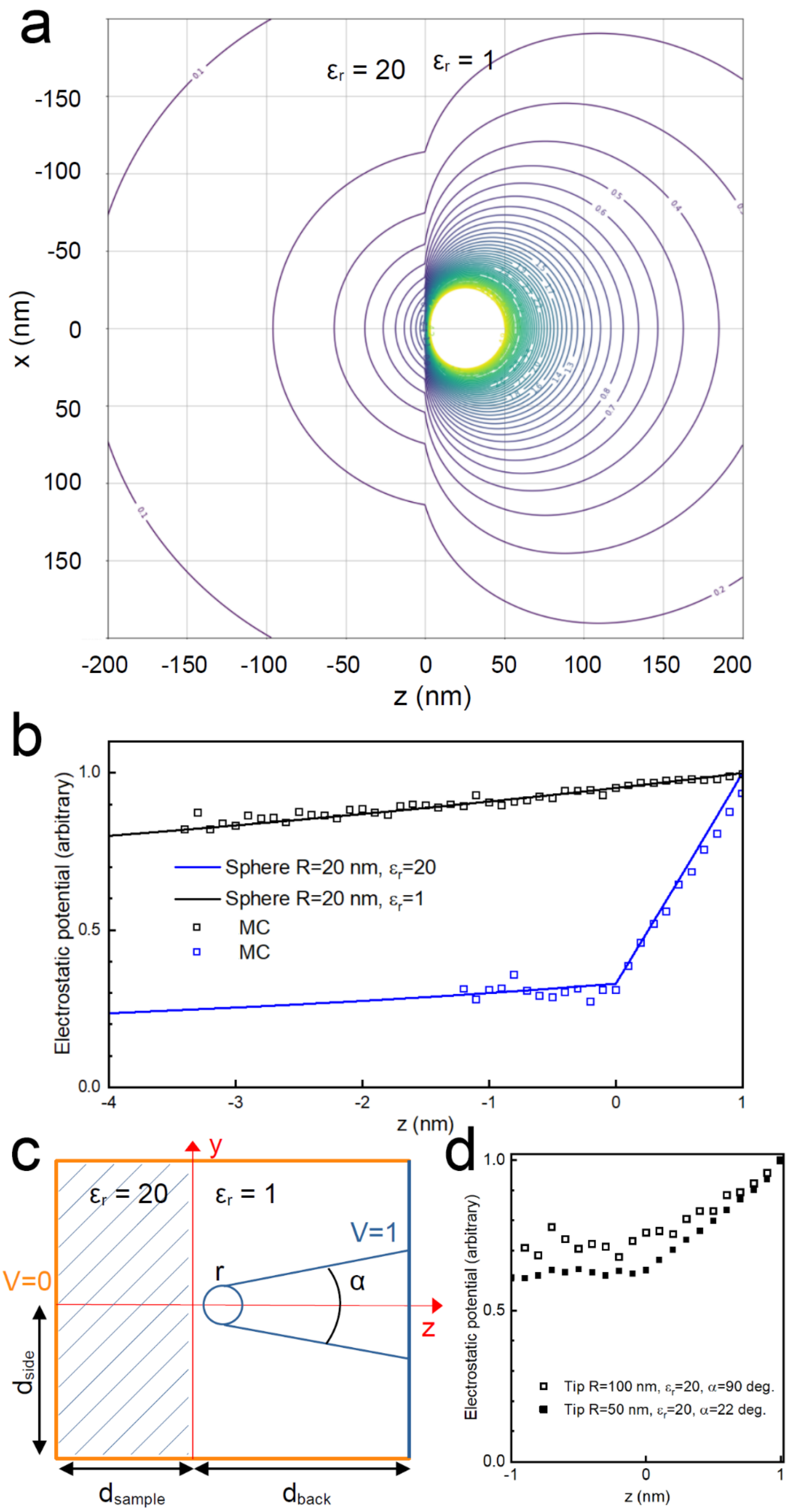

a
$\varepsilon_r$ = 20
$\varepsilon_r$ = 1
x (nm)
z (nm)
b
Electrostatic potential (arbitrary)
Sphere R=20 nm, $\varepsilon_r$=20
Sphere R=20 nm, $\varepsilon_r$=1
MC
MC
z (nm)
c
y
$\varepsilon_r$ = 20
$\varepsilon_r$ = 1
V=1
V=0
r
α
z
$d_{side}$
$d_{sample}$
$d_{back}$
d
Electrostatic potential (arbitrary)
Tip R=100 nm, $\varepsilon_r$=20, α=90 deg.
Tip R=50 nm, $\varepsilon_r$=20, α=22 deg.
z (nm)

***Figure S2. Effective bias in the tunneling junction.*** *a) Contour plot of the electrostatic potential of a point charge of +170 e placed 20 nm from an interface between vacuum and a sample with $\varepsilon_r$ = 20. b) Electrostatic potential along the symmetry axis in front of a sphere (R = 20 nm) with a potential of 1 (arb. u.), placed 1 nm from a sample with $\varepsilon_r$ = 1 and 20, respectively. Lines show analytical solutions, open squares represent MC floating random walk simulations. c) Sketch for modelling a realistic experimental configuration. d) Electrostatic potential in front of tips with various shapes, placed 1 nm from a sample with $\varepsilon_r$ = 20.*

## 1.5. Charge injection and field/light manipulation

Charge injection into hematite is achieved by electron/hole tunneling. The tunneling depends on the tip-sample distance *z*, and the potential barrier, which in turn depends on the LCPD and the effective bias (the potential difference between the tip and the active surface site in the tunneling process).

Discrete single-electron/hole injection events occur typically at a tip-sample distances 0.5 to 2 nm. At 3 nm, the tunneling barrier becomes so wide that no charge transfer occurred within the whole range of voltages (effective voltages of ±1.5 V). Holes require closer tip-sample distances than electrons due to the higher tunneling barrier. These distance values correspond to the distance between the last atom of the tip from the topmost plane of surface atoms. The tip-sample distance was estimated from the point where atomic resolution is obtained in AFM images (typically at tip-sample distances of 0.3 to 0.5 nm). The exact separation needed for the onset of single-charge tunneling depends on the bias value with respect to the Fermi level (*64*), modified by the electrostatic potential originating from the charges already injected to the surface. A higher absolute applied bias leads to enhancing the tunneling probability, but the charges injected into the lattice have a counteracting effect and eventually they limit further charge injection.

Figures S3a, b show an example of discrete electron injection into a 0.03 at.% Ti-doped hematite sample. The Kelvin parabola shifts towards more positive sample bias values, as electrons are injected. Single injection events are characterized by a stepwise increase in the frequency shift ($\Delta f$). The result is a time evolution with increasingly higher $\Delta f$ at very positive bias voltages (needed for electron injection), as shown in Figs. 1C for holes and S3a for electrons. In this work we generally assume that each step in the frequency shift corresponds to injection of a single polaron. We consider it unlikely that multiple polarons would form in a single step, because it is assumed in the literature that hematite favors single polarons; bipolarons (*65*) have not been reported. In principle it is possible that we miss out on a certain number of injected charges: The injected charges may diffuse away before the corresponding force can be detected in the AFM signal. This scenario does not seem to play a significant role because all steps in the polaron-injection curves have similar heights. This indicates that the charges injected to the sample form a polaron close to the surface.

The continuous or step-like decrease in $\Delta f$ after each injection event is attributed to polaron relaxations induced by the electric field of the tip and the electrostatic repulsion between injected charges. The electron polaron cloud shows remarkable time-stability at 4.7 K, as evidenced by Fig. S3b. Therefore, the continuous decrease in $\Delta f$ between single injection events (Figs. 1a and S3a) is predominantly induced by the tip electric field. In principle, the injection curves could be compared to the KMC simulations to obtain atomic-scale details about the involved processes, if measured and simulated at varying temperature and sample bias. Such analysis is beyond the scope of this work.

‘Quasi-continuous’ charge injection occurs when the tip is approached closer towards the sample. Figure S3c shows a frequency spectroscopy curve during tip approach. At the tunneling onset ($Z_{rel} \approx -7.4$ nm), the $\Delta f$ curve bends upwards until the area below the tip is ‘fully’ charged, i.e., charge injection is saturated at these specific distance and effective bias condition. The resulting LCPD shift is shown by the Kelvin parabolas in Fig. S3d. Here, it is important to note that upwards bending of the $\Delta f$ curve (red part in Fig. S3c) is induced by the rapid injection of multiple charges and not by a traditional force-distance curve describing a repulsive interatomic interaction potential (the tip–sample distance is too large for repulsive interaction).

The applied potential between the tip and the sample induces an electric field that can interact with the polarons in the material. Figure S3e shows the time evolution of the frequency shift when the tip is located ~100 nm above the sample (far from tunneling conditions) and the surface is illuminated by a light source (3 W white LED diode placed outside the UHV chamber). The effective applied sample bias was -1.6 V. When the light is switched on, photogenerated electrons are attracted towards the tip, increasing the surface potential to compensate the electric field inside the sample. Figure S3f shows modifications of the LCPD induced by applying a ±1.6 V potential under light illumination. The shape of the cloud reflects the electric field, and therefore the mesoscopic shape of the tip.

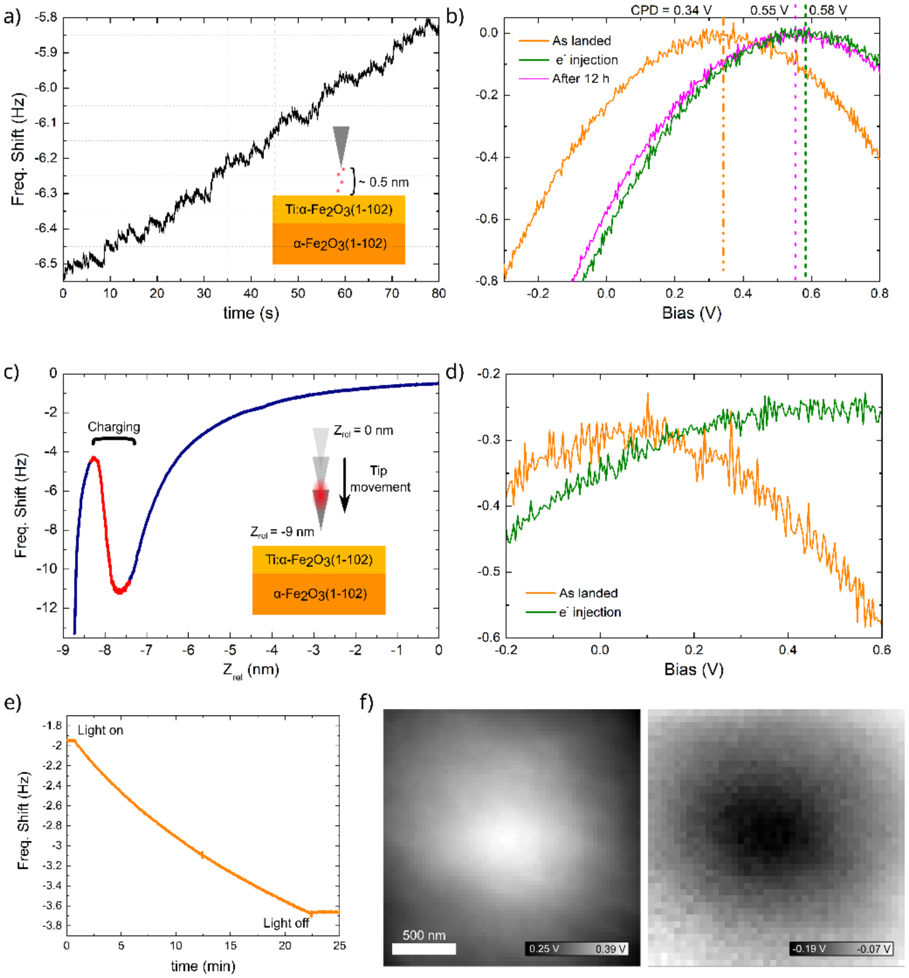


***Figure S3. Electron injection.*** *a) Single electron injection events ≈1 nm from the surface at +0.8 V and T = 4.8 K. The inset shows evolution of the frequency shift, after injection of ≈100 electrons. b) Kelvin parabolas of the surface before and after injection of ≈300 electrons at a maximum effective sample bias of +1.2 V, measured at a tip-sample distance of ≈5 nm. The orange 'as landed' parabola was obtained before injection. Green and pink parabolas show the LCPD immediately after e- injection and 12 hours later. c) Frequency-shift vs. z-spectroscopy. The red region marks the shift of the Kelvin parabola due to continuous charging at +1.2 V. d) Kelvin parabolas before and after the continuous-charging experiment in (c). e) Time-evolution of the frequency shift when the light is switched on and a sample potential of +1V is applied between tip and sample, out of the tunneling conditions at a distance of 3 nm. f) Electron and hole polaron clouds created by the combination of light illumination and applied bias of -1.6 and +1.6V for left and right panels, respectively. The tip was out of the tunneling conditions, approximately 100 nm far from the sample.*

## 1.6. Imaging the potential after single hole injection events

Atomic resolution of hematite is incompatible with single-polaron identification: Atomic resolution requires low tip-sample distances where tunneling is facile, thus the charge distribution would be modified. However, single polarons can be localized by the electrostatic field they induce. Figures S4 a,b show constant-height images before and after controlled single hole injections. Both images were acquired using the same scanning parameters (size and scan velocity, tip-sample separation, bias voltage, and oscillation conditions). The injection was carried out by approaching the tip at five different positions until step-like changes in frequency shift were detected (details are shown in Fig. S5). The yellow triangles

in Fig. S4b indicate these tip positions and the numbers of holes injected. Figure S4c (the same as Fig. 1E) is the result of subtracting the images b) and a). Figure S4d is a color-coded 3D representation of the c) for better visualization of the hole clouds.

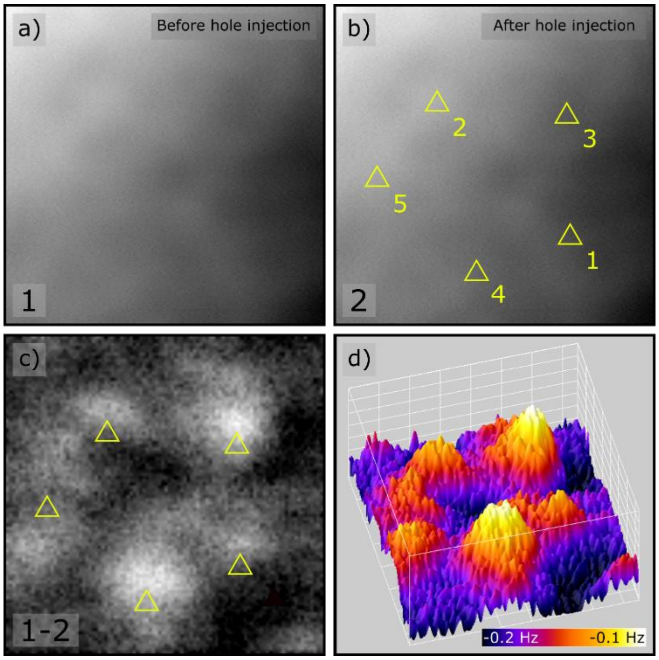


***Figure S4. Imaging single polarons.*** *a) and b) constant-height scans before and after single hole injection experiments. Both images were acquired at −0.7 V (the LCPD was +0.8 V) at a tip–sample distance of ≈3 nm to ensure that tunneling cannot occur. The yellow triangles show points where defined numbers of hole-polarons were injected; the number of holes is marked by the respective numbers. The image size is 30 × 30 nm$^2$. c) Difference image of panels a) and b). The image has a subtracted linear background to increase the contrast. d) 3D view of c).*

To the eye, the two maps of the potential in Figs. S4a,b show only marginal changes. However, the difference between both images results in clouds close to the positions of the charging events, see Fig S4c. The clouds for one and two injected holes show an apparent increase of $\Delta f$ ≈0.05 Hz w.r.t the background. The estimated radii for one- and two-hole clouds are ≈3 and 4 nm, respectively, and their size is mainly attributed to the finite tip radius and a larger tip-sample distance of 2-3 nm. Performing this experiment with different tips led to various radii of the features, ranging from 3 to 10 nm. The detection is based mainly on the long-range electrostatic forces that decay as ~$1/r^n$, therefore the tip radius and the tip-sample distance have a pronounced effect on the spot size. The clouds for 3 and 4 injected holes show an increase in $\Delta f$ of ≈0.1 Hz w.r.t the background and a radius of ≈4.5 nm and 6 nm, respectively. The five-hole cloud has a ≈0.05 Hz increase w.r.t to the background and a ≈7 nm radius.

Details of the hole-injection processes relevant for Fig. 1E and S4 are shown in Fig. S5. The plots show the evolution of the frequency shift over time; the events corresponding to hole injections are marked by blue arrows. The last, five-hole panel also contains step-down events, marked by green arrows. These indicate hopping of the holes over several unit cells. Hopping over several unit cells is consistent with the shape of the corresponding cloud in Fig. S4c spreading out over a larger area and appearing weaker than the four-polaron cloud.

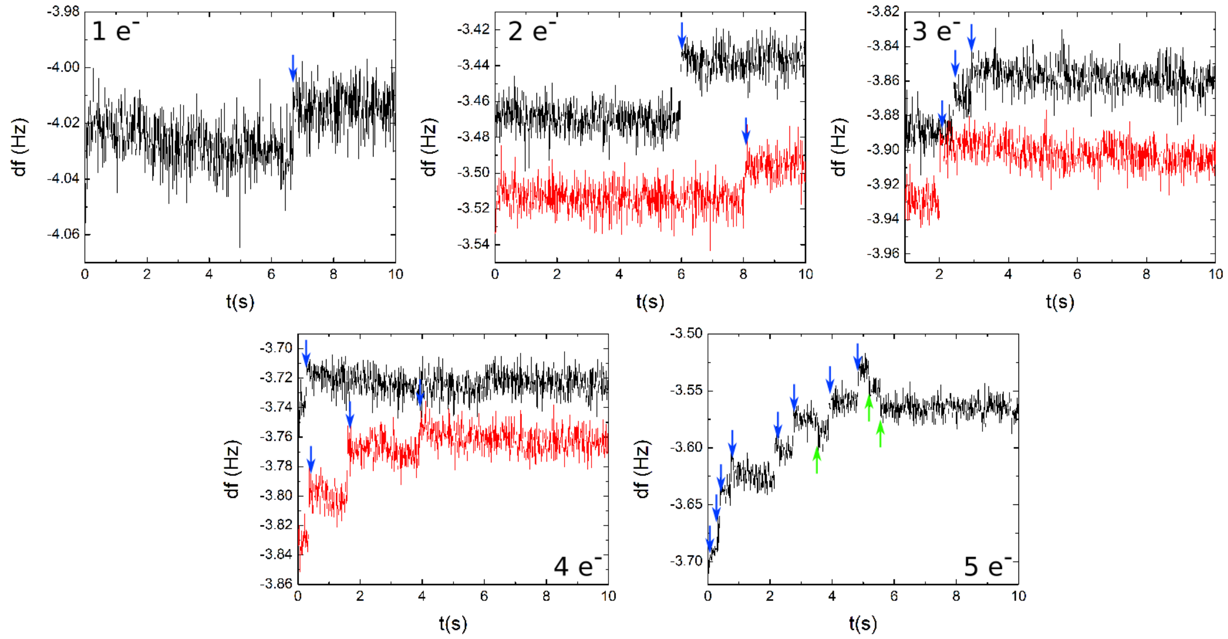


***Figure S5. Detail of the hole-injection events from Fig. S4.*** *The curves show the evolution of the frequency shift when holes are injected into the surface. When multiple curves are displayed in one plot, the black was curve was measured before the red one and the red curve typically used a slightly closer tip-sample distance or higher absolute value of the tunneling bias. Single-hole events are marked by blue and green arrows for injection and relaxation, respectively.*

## 2. Kinetic Monte Carlo simulations

### 2.1. Temperature effect on polaron clouds and effects of doping

The polaron clouds formed after charge injection have a circular symmetry (such as Fig. 3A or Fig. 4A); no significant deviation from this shape has been noticed throughout all experiments. An exception are cases where the anisotropic cloud shape apparently originates from an asymmetric tip, see the profiles in Fig. S6a for instance. The circular symmetry of the clouds indicates an almost isotropic migration of polarons. The delocalized transition state found in our DFT can explain this behavior.

Figure S6 shows the evolution of the electron-polaron line profiles with increasing temperature for natural, 0.03, 0.7 and 3 at. % Ti-doped hematite samples (the data for 0.03 and 3% in Fig. S6b,d are identical to Fig. 3B,C in the main text). As the doping level increases, the temperature required to scatter the cloud decreases. The evolution of the profiles carries information about bulk vs. surface diffusion of polarons. As detailed by the KMC results in the section '2.4. Surface vs. Bulk Migration', surface migration results in spreading of the cloud, *i.e.*, the profile becomes wider, while bulk migration results mainly in a decrease of the overall profile height. While a detailed analysis needs to consider the tails of the profile, which are difficult to measure precisely, comparison with the simulations in Fig. S9 indicates that the experimental electron-polaron data are incompatible with migration limited to the surface layer. The highest-doped samples (panels c and d in Fig. S6 and Fig. 3C) indicate migration limited to the thin Ti-doped film; here a characteristic feature is that the high-temperature curves overshoot the low-temperature curves at large distance from the center of the cloud. This agrees with our analysis showing that the electron mobility is higher in the Ti-doped films than in the undoped (bulk) hematite substrate used for the film growth. The low-doped samples (panels a, b in Fig. S6) indicate bulk migration of electron polarons, because the electrostatic potential in the center of the cloud decreases, and the shoulders far from the center overlap.

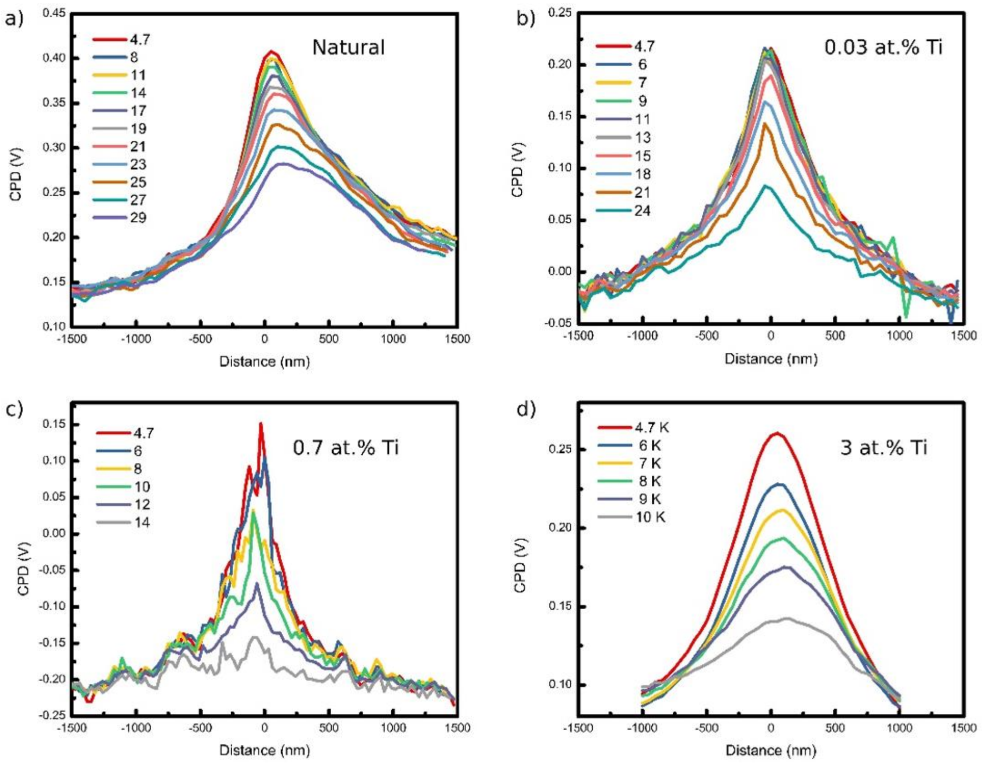


***Figure S6. Evolution of electron-polaron clouds with the temperature,*** *for different levels of Ti doping. The panels show a decay of the clouds with the temperature, in samples doped by 0% ('natural'), 0.03%, 0.7% and 3 at.% Ti. Note that the panels use different color schemes for the temperature.*

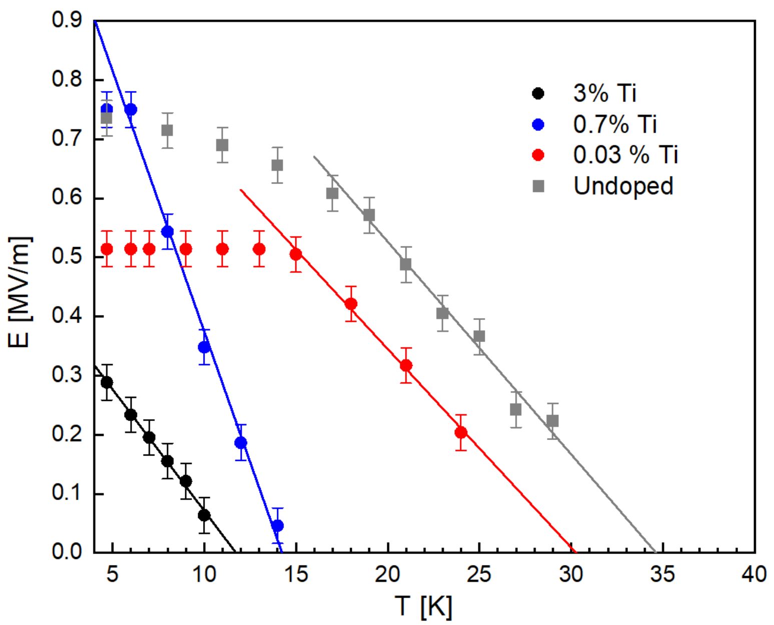


***Figure S7. Analysis of electric field vs. temperature inside clouds of electron polarons.*** *The highest fields in the cloud were estimated from Fig. S6 and plotted as a function of temperature for samples doped by 0%, 0.03%, 0.7% and 3 at.% Ti.*

The data from Fig. S6 can be processed analytically, see Fig. S7. Polaron migration is activated by a combination of thermal excitations and the electric field originating from electrostatic repulsions among the polarons. The field can be estimated by differentiating the LCPD profiles in Fig. S6; the value is zero in the center of the cloud and has a maximum value close to the edge of the region where the polarons physically reside. This edge corresponds to the highest slope of the electrostatic potential. Outside the cloud, the potential decays approximately as 1/x, according to Gauss's law. The polaron diffusion is therefore most active in the region with the highest electric field. It is interesting to plot the highest in-plane electric field as a function of the temperature, see Fig. S7. The fields were estimated by a linear fit of the steepest part of the LCPD profiles in Fig. S6. The data in Fig. S7 therefore provide an estimate for the electric filed in the x-y plane. Notably, these values may be underestimated because the experimental LCPD data are measured in certain distance above the surface (effectively 10-100 nm, depending on the tip sharpness).

Fig. S7 shows that for all Ti doping levels, the maximum field decays linearly with temperature in the higher-temperature part of the curve, and some doping levels show a flat plateau in the low-temperature regime (0 and 0.03% Ti). The rather slow decay, as compared with a typical Arrhenius behavior, is related to the combined action of thermal excitation and the electric field. The polaron hopping is activated easily in the high-field regime and ceases when the field is reduced by reorganization of the polaron cloud. Some of the doping levels show only a weak temperature dependence in the low-temperature region (undoped and 0.03% Ti). This region is caused by the fact that the initial polaron distribution is formed by the tip electric field, which 'pushes' the polarons into the material. For the undoped sample, some polaron spreading occurs even in the low-temperature region. This may indicate certain polaron mobility that is not linked to thermal excitations; this mobility may be either induced by residual radiation (infrared or visible) in the cryostat, or from tunneling of polarons (*66, 67*).

The linear fits can be extrapolated to the value of zero electric field, i.e., crossing the x-axis. This occurs at temperatures given in Table ST2. From this temperature $T_0$, it is possible to make an approximative estimation of the activation energies for polaron hopping, assuming that the hopping rate at this temperature is in order of the time used in the sample-annealing cycles, *i.e.*, $10^{(0\pm2)}$ $s^{-1}$: $E_A = k_B T_0 \ln(v_0)$. The resulting activation energies are given in Table ST2, using a fixed prefactor of $10^{13}$ $s^{-1}$. The results estimated within this simple approximation are well in-line with the values obtained by a full KMC simulation in the main text (Table 1).

| Ti doping level | $T_0$ [K] | $E_A$ ($v_0$=$10^{13}$$s^{-1}$) |
|---|---|---|
| 3 % | 11.7 | 30±5 meV |
| 0.7 % | 14.2 | 37±6 meV |
| 0.03 % | 30.3 | 78±12 meV |
| Undoped | 34.6 | 89±14 meV |

***Table ST2: Activation energies for electron-polaron hopping****, estimated from the zero-filed limit in Fig. S7.*

## 2.2. Other models considered for Kinetic Monte Carlo simulations

Besides the model described in the methods section of the main text (hereafter denoted M1), additional two models M2 and M3 were tested (see details below).

M2 - model with suppressed electrostatic interaction. We argue that the experimentally observed LCPD map as a function of temperature is the result of electrostatic repulsion between the polarons. To confirm this statement, we have simulated the cloud evolution by thermal diffusion without the electrostatic interaction. The potential above the point of injection scales with the inverse of the mean diffusion path, and thus as $\sqrt{\nu}\ \ exp[-E_A/2k_BT]$. In this case, even lower values of the prefactor, ~$10^3$ $s^{-1}$, are needed to protect the charge carriers from freezing at low temperatures and extremely rapid diffusion at higher temperatures.

M3 - model with locally decreased hopping barrier in the proximity of dopants. The uniform value of the diffusion barrier in the M1 model, neglecting the *local* interaction of the polarons with dopants is evidently a simplification. In a more realistic picture, the dopants locally influence their surroundings. In the first approximation of the local influence, we reduced the barrier by a fixed value $\Delta E_{loc}$ at a distance $r < R$ of the randomly placed dopants. The value of barrier at a distance $r > R$ from any dopant was set to the value obtained by fitting the polaronic cloud in the nominally undoped sample, $E_A^{0\%}$, using M1. In the case of highly doped sample (3% Ti) we expect nearly all sites to be affected by the dopants, allowing to estimate $\Delta E_{loc}=E_A^{0\%}-E_A^{3\%}$. This adds to the model M1 a single parameter *R,* independent of the dopant concentration. However, it was impossible to find the value of *R* that results in a reasonable agreement with the experiment for all studied dopant concentrations. Building more complex rules for the local effect of the dopant on the diffusion barriers can probably get a better fit, but at the cost of additional parameters.

In model M1, several additional aspects have been neglected. First, an unknown amount of oppositely charged polarons from the surroundings may be expected to get attracted by the injected charge cloud. These polarons likely recombine with the injected ones when their mobility is increased by annealing. Neglecting this process can be the reason for the experimentally observed lowering of the potential at tails of the LCPD curves, compared to KMC simulations (see Fig. 3B and C in the main text). Second, the relative permittivity of hematite is slightly anisotropic (*68*), might depend on temperature (*64*) and can be influenced by doping. We assume these variations are not substantial and usage of the single value of the relative permittivity selected in the range of theoretically (*68*) and experimentally (*67*) obtained values of static relative permittivity $\epsilon_r$ = 20 not to qualitatively affect the results.

## 2.3. Overview of KMC simulations with different parameters.

In the KMC simulations, three parameters were considered: The frequency prefactor $\nu_0$, the activation energy $E_A$ for activating the polaron migration (in the absence of the electric field from other charges), and the hopping distance *d*, which mainly affects the impact of the electric fields. Fig. S8 provides an overview how the simulated LCPD profiles depend on these parameters. The columns show results that aim to match experimental data measured at samples with the respective Ti doping levels. Rows represents various values of the parameters $\nu_0$ and hopping distance *d*. The first four rows are marked as 'Lattice model'; here the polaron migration proceeds in a fixed cubic lattice. The last two rows are marked as 'Off-lattice model', which means that the polaron migration is not restricted to discrete lattice positions. This model is suitable for longer hops, where a fixed cubic with a lattice parameter as large as

the hopping distance would induces artifacts. The used value of $E_A$ is marked in each plot; the value was chosen to obtain agreement for the highest-temperature curve in the set.

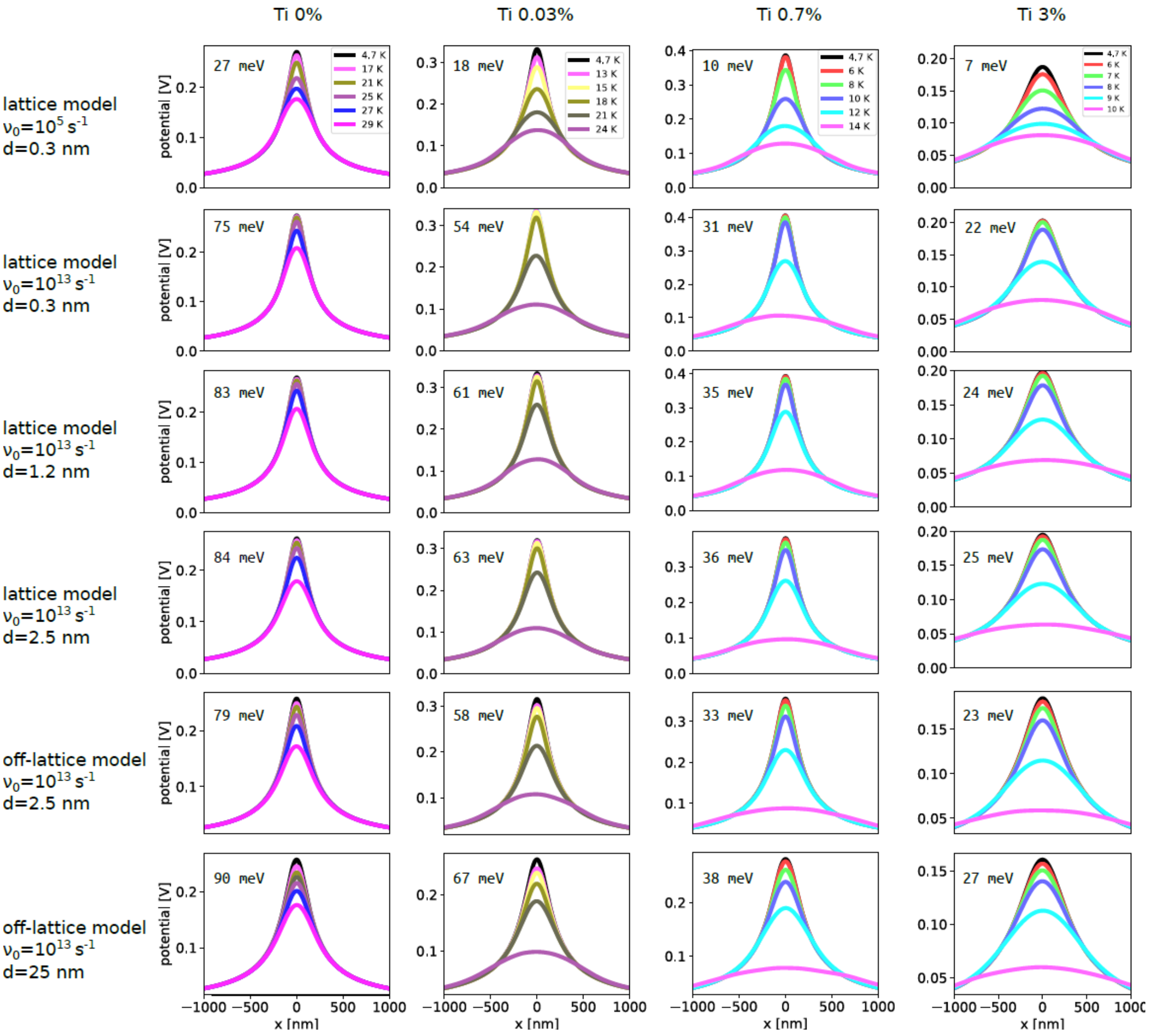


***Figure S8. Simulated LCPD profiles calculated 50 nm above the surfaces.*** *The simulations aim to match experimental data of electron-polaron hopping measured for different Ti doping levels (as in Fig. S6; the respective Ti doping levels are marked for each column). The rows show simulation results for various parameters and KMC models, for details see the text.*

One way to achieve agreement with the experiment is the assumption of polaron hopping over interatomic distances (0.3 nm, isotropic), but using a low frequency prefactor of $10^{-5}$ $s^{-1}$ (the first row in Fig. S8). This is 8 orders of magnitude difference from the standard value of $10^{13}$ $s^{-1}$, derived from the frequency of lattice vibrations. While the match between KMC and experiment appears excellent, we consider this scenario unphysical, since such a low prefactor is hard to justify. Below we discuss several mechanisms that might be responsible for the low prefactor and argue why these options do not match

quantitatively: (a) polaron tunneling, (b) a high entropy of the transition state, and (c) hopping in a spatially restricted region.

(a) It can be stated that the low prefactor does not originate from (quasi)particle tunneling (*69*). A characteristic feature of tunneling is that the low-temperature region contains certain contribution from temperature-independent mobility and thermally-activated processes take over at higher temperatures. In principle, when fitting data from the border between thermally excited hopping and tunneling-induced hopping, one could obtain erroneous conclusions about the frequency prefactor. The low prefactors in our case do not originate from this effect: Only data from the undoped sample show some temperature-independent mobility (Fig. S7), and this would not dramatically influence the result of the KMC fitting.

(b) Second, the experimentally measured prefactors may include an entropy contribution (*70*) that originates from entropy difference between the initial and the transition states. The hopping rate is given by the change of the Gibbs energy between the initial and the transition states:

$$\nu = \nu_0 e^{-\Delta G/kT} = \nu_0 e^{\frac{-\Delta E + T\Delta S}{kT}} = \nu_0 e^{\frac{\Delta S}{k}} e^{\frac{-\Delta E}{kT}}$$

If there is any entropy difference between the transition and the initial states, it appears as a change in frequency prefactor in experiments. In our case, the estimated prefactor of $10^5$ $s^{-1}$ deviates by 8 orders of magnitude from the expected value, which would correspond to an entropy change of $k_B \ln(10^8) \simeq 18\ k_B$. Translating this value to the lattice vibrations (phonons) (*71*), the low-temperature specific heat per atom in the Debye model is $C = 234\ k_B(T/T_D)^3$, where the Debye temperature for hematite is 490 K (*1*). This corresponds to an entropy of $S = 78\ k_B(T/T_D)^3$ per atom. Assuming T=20 K, the vibrational entropy per atom is 0.0053 $k_B$. The transition state would therefore need to completely immobilize more than 3500 lattice atoms in the transition state, which appears unrealistic. This option therefore cannot quantitatively explain the magnitude of the prefactor (even though, it may partially contribute).

(c) The polaron migrates in a restricted area and only some sites in this region allow for transition out of the area. This mechanism is known to provide low frequency prefactors (*72*) and is consistent with the picture of polarons that migrate in the vicinity of a Ti dopant. This appears unlikely, however, because the prefactor should depend on the doping level, which is not observed. Further, the polarons should quickly relax to the lowest-energy sites and the prefactors would converge to the regular values (*73, 74*).

In summary, we do not see any physical justification for a pre-exponential factor as low as it comes from fitting a simple model with polarons hopping to nearest-neighbor sites. In addition, a nearest-neighbor-hopping model is also ruled out by the absence of a strong anisotropy in our LCPD maps, as it would appear due to the anisotropy between hopping in the basal plane and across the planes of equal spin direction. (Note that the basal plane intersects our surface at an angle of 53.7°; easy hopping in the basal plane should be observable as fast spreading along the [1 1 −2 0] direction and slow spreading along [1 −1 0 −1].)

## 2.4. Surface vs. bulk migration

Fig. S9 shows the difference between 2D and 3D migration of polarons. The left panel shows the case where bulk migration is allowed, the right panel shows migration restricted in the 2D surface layer.

The middle panel shows an intermediate case where the migration is restricted to the thin film of Ti doped material. The main difference lies in the 'tails' of the potential profiles: In the case of a pure surface migration, the tails simulated for higher annealing temperatures are higher than the curves measured after annealing to lower temperatures. The same effect does not occur for bulk migration: Here the annealing results in a decay of the curve in the whole range. Note that the magnitude of the curves is comparable in both cases. The experimental data in Fig. S6 are mostly consistent with bulk migration. The only exception is the 3% Ti doping, where migration restricted to the 2D film provides a better fit.

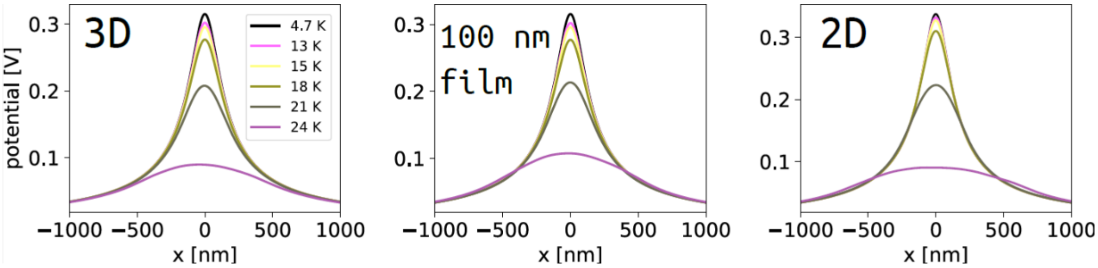


***Figure S9. Surface vs. bulk migration.*** *LCPD profiles calculated 50 nm above the surface, considering bulk migration (left), migration confined to the thin Ti-doped film (middle) and purely 2D migration at the surface (right). The simulation parameters for 0.03% Ti doping were used.*

## 2.5. KMC simulations of hole hopping with consideration of trapping centers

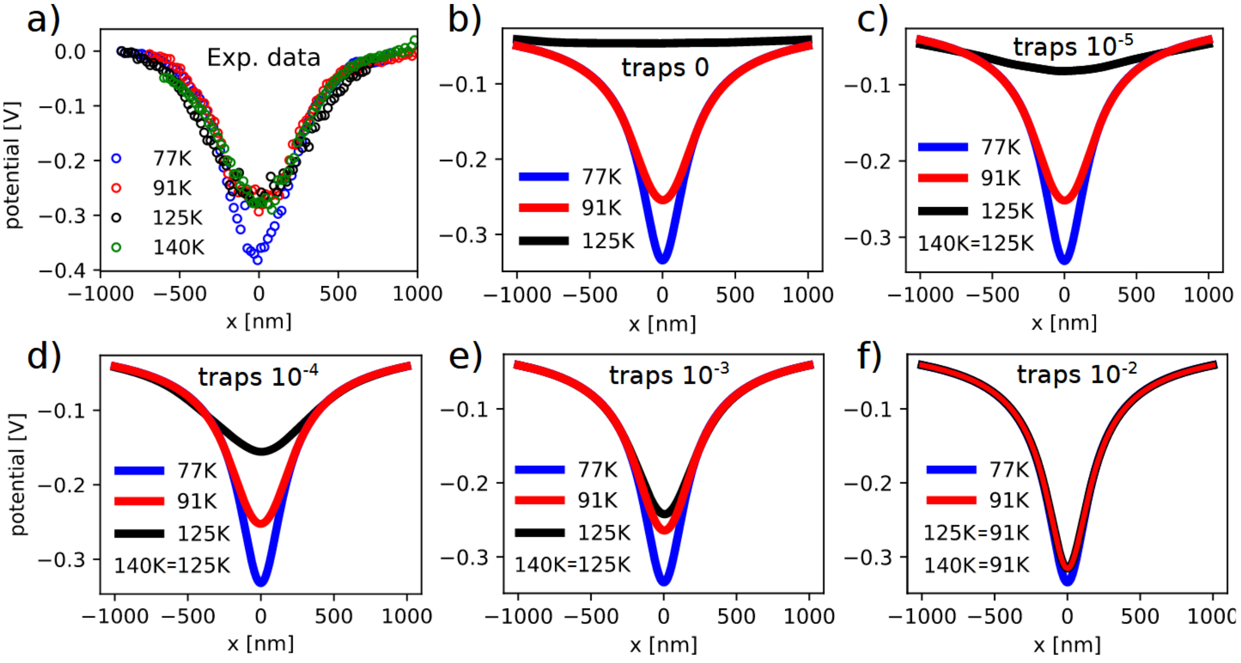


***Figure S10. Hole polarons and trapping centers.*** *LCPD profiles calculated 5 nm above the surface. The simulations aim to match experimental data of hole polarons in the sample doped with 0.1% Ni. a) Experimental data. b-f) Simulated profiles of the electrostatic potential for various trap concentrations.*

Fig. S10 shows the KMC-simulated LCPD profiles calculated above a cloud of holes for different concentrations of randomly distributed traps, compared to the experimental data. Polarons reaching a trap are immobilized for the rest of the simulation. The model parameters are first fitted for 77 K and 91 K in the case without traps (Fig. S10b). Then, annealing to 125K results in complete dissolution of the polaron cloud, in contrast to the experimental observation (Fig. S10a). The best agreement has been achieved for a trap concentration of $10^{-3}$ (Fig. S10e). A further increase of the trap concentration results in faster ‘freezing’ of the LCPD, as shown in Fig. S10f.

The concept of hole-traps is well established in photocatalysis. One can speculate about the origin of such trapping centres: (i) They can be defects present in the lattice before the polaron injection. It is noteworthy that the calculated concentration of $10^{-3}$ matches the level of Ni doping. (ii) The holes can weaken the lattice oxygen bonds and promote defect formation. iii) The experiments were performed on a p-doped layer grown on a bulk crystal, which is presumably slightly *n*-doped due to oxygen vacancies. The initial decay of the hole-cloud could be due to the hole annihilation by underlying electrons, which stops later due to the formation of a *p*-*n* junction.

# 3. DFT calculations

## 3.1. Electronic occupation in hematite

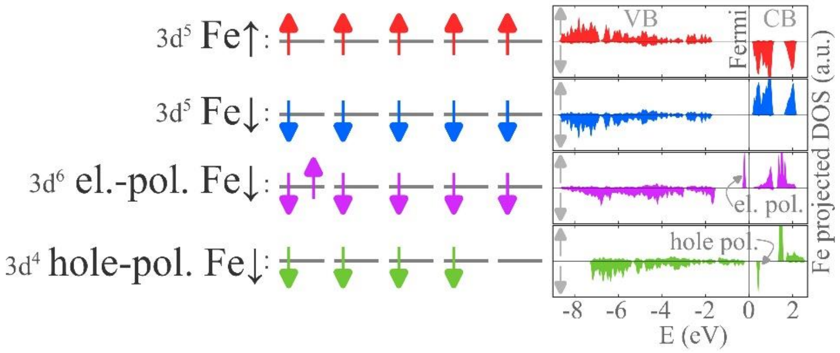


***Figure S11. Sketch of the occupation of the 3d energy levels in hematite***. *Fe sites show fully filled majority spin channels and completely empty minority spin channels, antiferromagnetically ordered (see Fe↑ and Fe↓). The electron polaron localization occurs on the minority spin channel, while the hole removes one electronic state from the majority spin channel.*

In our spin-polarized DFT calculations, hematite $Fe_2O_3$ can be viewed as a stack of layers (referred to as planes for simplicity) of ferromagnetically ordered Fe atoms with (practically) fully filled majority and completely empty minority d-orbital spin channels. Therefore, electron polarons can only localize on the minority spin channel, while hole polarons localize on the majority spin channel (the only channel with available states). Figure S11 sketches the occupation on every plane and shows the corresponding density of states of a reference Fe atom.

### 3.2 Ti doping

It is important to notice that every Fe plane is sandwiched between two planes with opposite spins. From this simple picture, polaron hopping can only occur between planes with the same ferromagnetic alignment, since there is no available state for the polaronic state to form on the Fe atoms with opposite alignment. Therefore, a hopping-based polaron diffusion mechanism must show a strong anisotropy.

Experimentally, we have found a significant enhancement of polaron mobility upon Ti doping, already at concentration as low as 0.1%, *i.e.*, one Ti dopant in a volume of 10×10×10 Fe atoms. We performed DFT calculations on doped hematite: In our setup, the substitution of one Fe atom with Ti corresponds to a low doping concentration of ≈0.5%. Results are summarized in Figures S12–S14.

The Ti substitutional doping introduces an excess electron that is predicted to localize on an adjacent Fe atom, since the Ti itself is not a good trapping site. Moreover, charge localization around the Ti site is not particularly strongly favored by the defect (similarly to recent studies on Si doping): [(40)] One particular pair of Fe atoms stabilizes the electron polaron by up to 50 meV more favorably as compared to other sites around the defect. This favorable site is shown as the yellow isosurface in Fig. S12A.

In addition to the intrinsic excess electron introduced by the Ti dopant, we modeled the system by adding an additional electron, mimicking the injected electron in the experimental measurements. The injected electron (blue isosurface in Fig. S12A) can localize on the remaining sites (i.e., atomic sites available for localization besides those hosting the Ti-induced electron polaron), and it is associated to an in-gap state deeper in energy by only 20 meV as compared to a polaron in the pristine system (see DOS in Fig. S12B). In addition to the small electronic energy gain for localization near the Ti, the energy cost to distort the lattice is increased by the Ti defect. The cost to distort the pristine structure to accommodate one polaron is 335 meV, while it raises to 345 and 360 meV for electrons surrounding the Ti defects (the yellow and blue isosurfaces in Fig. S12A, respectively). Moreover, the polaronic distortions at the equilibrium appear different: for instance, in the pristine system the Fe-Fe distance on the hosting sites is 2.77 Å, while on the sites surrounding the Ti dopant is ≈2.82 Å (a value closer to the distance of 2.98 Å of the delocalized solution).

In pristine hematite, we were able to model in our DFT calculations the system hosting excess electron(s) in either polaronic or delocalized state(s). Therefore we are able to calculate the polaron formation energy, $E_{POL}$(pristine) = −56 meV, by using the standard definition, i.e., the formation energy is obtained as the difference between the energy of the system hosting polaronic states, and the energy of the system with delocalized electrons. Conversely, in the presence of Ti doping, we are unable to obtain the delocalized-electron solution in our DFT calculations. This is most likely due to the smaller polaronic distortions in the doped system (described in the paragraph above) and the symmetry breaking due to the Ti: Although we inizialize the system in the delocalized solution, the electronic and ionic relaxation converges to solutions with localized charges only. Thus, we are unable to calculate the polaron formation energy in the doped system using the standard definition.

However, we can obtain an upper-limit estimation of the polaron formation energy using the following strategy. We modeled the Ti-doped hematite removing an excess electron from the system and relaxed the lattice to its equilibrium structure. Then, we reintroduced one electron and constrained the ions to the positions obtained in the previous step. In this way, we were able to obtain a constrained solution for the delocalized state, which obviously introduces a fictitious energy penalty. The results are

reported in Table ST3. In this setup, the polaron formation energy is $E_{POL}$(Ti) = −145 meV: the polaron appears much more stable than in the pristine system, which might be considered an artifact of the constrained calculation penalizing the delocalized solution.

Rather than the direct comparison with the pristine system, it is more interesting to analyze the trend of the polaron formation as more electrons are injected into the system. In the pristine system, the energy stability increases while going from one to two polarons: the average formation energies changes from −56 meV to −71 meV per polaron. Conversely, Ti dopants seem to disfavor the formation of additional injected electrons: the average polaron formation energy weakens from −145 meV for the single (intrinsic-Ti) polaron to −139 meV per polaron in the case of two excess electrons.

| (*=constrained) Polaron Formation Energy | pristine hematite | | | Ti-doped hematite | | |
|---|---|---|---|---|---|---|
| | 1st polaron | 2nd polaron | average | 1st polaron | 2nd polaron | Average |
| 1 excess electron | −56 meV | – | −56 meV | −145 meV* | – | −145 meV |
| 2 excess electrons | −56 meV | -85 meV | −71 meV | −145 meV* | −133 meV* | −139 meV |

***Table ST3: Polaron formation energy for the pristine and Ti-doped system****, including one or two excess electrons. In the Ti-doped case, the delocalized solution was obtained by constraining the lattice structure to equilibrium position of the analogous system with no excess electron: thus, these energies (marked by the * symbol) represent an upper limit of the polaron formation energy. In the case of two electrons, the individual polaron energies were assigned by considering the 1st polaron unperturbed (i.e., we assigned to this polaron the same energy as obtained in the one-electron case).*

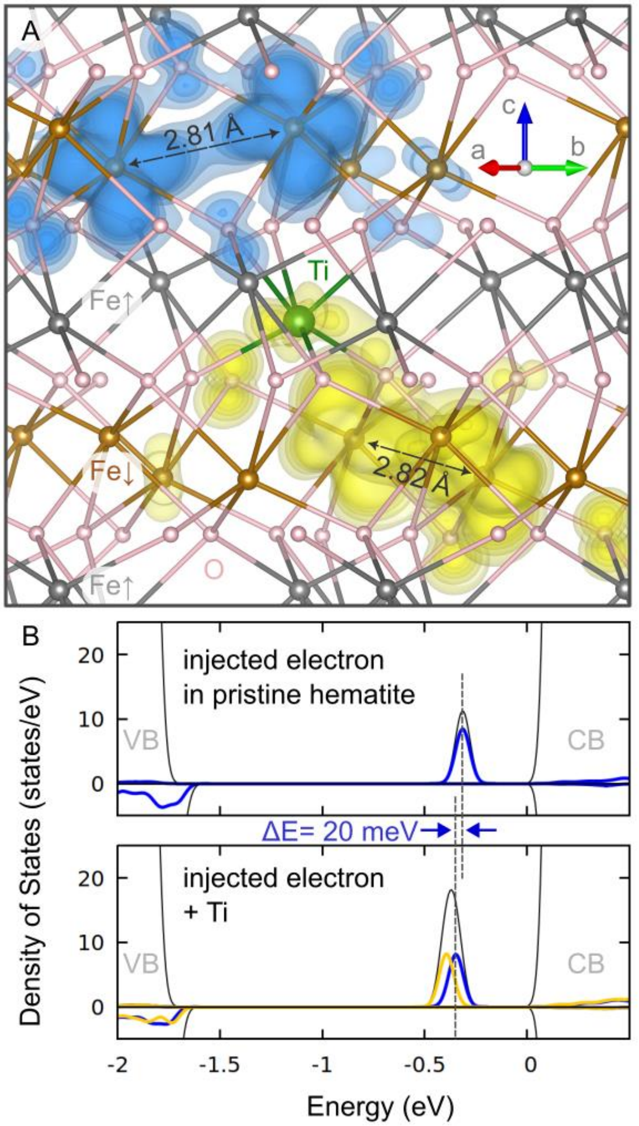


***Fig. S12: Ti doping.*** *Panel A shows the spatial distribution of two localized electrons (yellow and blue isosurfaces), one introduced by the Ti dopant and one representing the electron injected by the tip. O atoms are shown in pink, Ti in green, Fe in brown and grey to distinguish between opposite orientations of the local magnetic moments. Panel B shows the corresponding density of states for the two localized electrons (in yellow and blue, respectively) as compared to the case of one injected electron in the pristine hematite (bottom and upper graphs, respectively).*

## 3.3 Diffusion of electron-polarons in DFT

The polaron diffusion within one ferromagnetic Fe (0001) plane is well described by the hopping mechanism between adjacent sites, where the initial, final and all transition states are polaronic (Fig. 2 in the main text). Conversely, polaron hopping across (0001) planes is hindered by the Fe atoms in the adjacent plane, which have the opposite spin and thus, no empty state in the spin channel of the polaron. Along this direction, the Ti dopant seems to play a crucial role. Figure S13 shows the charge density distribution of the initial and final polaron states in the inter-layer hopping. In the undoped crystal, the polaronic state is confined in the ferromagnetically ordered (0001) Fe plane (and on the neighboring oxygen atoms, see Figs. S13A,B). As reported also in Table ST4, no sizable polaronic charge occupies the

Fe atoms in the adjacent plane due to the antiferromagnetic order. In the presence of the Ti dopant (Figs. S13C,D), there is a stronger overlap of the wavefunction of the initial and final polaronic states, which favors the polaron hopping process. (*64*) Therefore, the Ti dopant acts as bridge in the electron polaron diffusion across antiferromagnetically ordered Fe planes; it provides empty states in the spin channel of the polaron. Fig. S14 shows the effect of Ti in the pure polaron-hopping picture (here we model one polaron hopping in the proximity of Ti, as depicted in Fig. S14E,F). The hopping barriers for both the in-plane and inter-plane hopping are reduced by the Ti doping to a small extent (Fig. S14A and B). The DOS plots in Fig. S14C and D show that the evolution of the polaronic states during the hopping is not qualitatively altered by the Ti dopant as compared to the pristine case (Fig. 3 in the main text). These results show that Ti dopants facilitate inter-plane diffusion, albeit to a lesser degree than required for the large enhancement of the mobility observed by the experiments. Another restriction is that the facilitated inter-plane diffusion occurs only for a single polaron, i.e., the polaron stemming from the charge donated by the Ti. In our case, where an extra electron is injected by the tip, two polarons will localize at the Ti as shown in Fig. S12; preferably at opposite sides of the Ti, impeding the interlayer diffusion path for each other. Most importantly, as discussed in the main text, the simple polaron-hopping picture fails to describe the experimental observations, and we conclude that the diffusion occurs via a transient free-electron-like state, where the electron motion strongly depends on the external electric field. Facilitating interlayer hopping as shown in Fig. S14 therefore does not explain the increase of mobility with Ti doping.

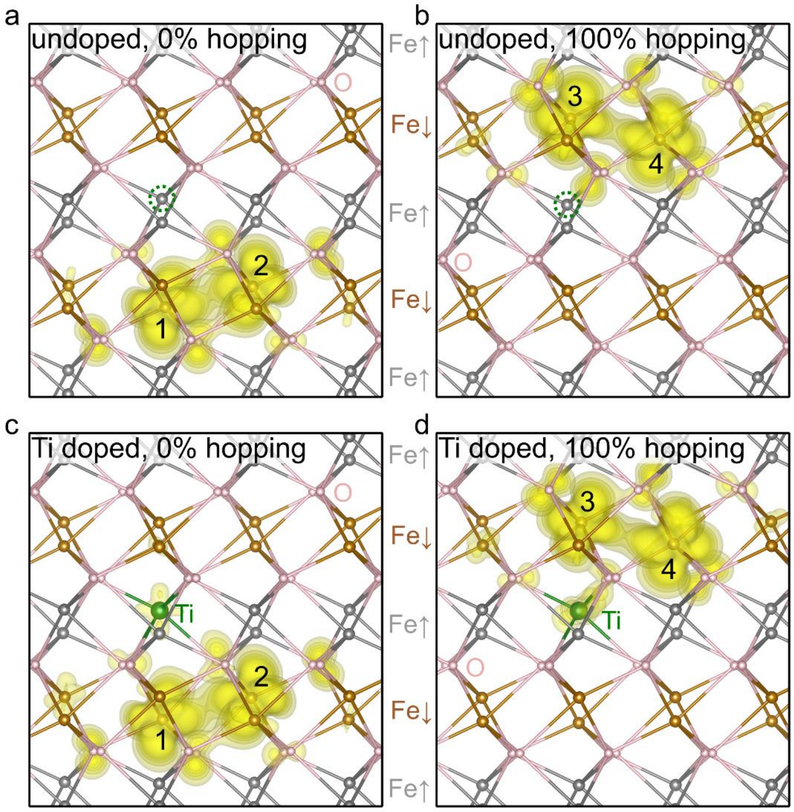


***Fig. S13: Initial and final electron polaron localization in the inter-layer hopping process.*** *The panels show the initial (0%; a,c) and final (100%; b,d) hopping states in Fig. 4B in the main text. Panels a,b and c,d refer to the undoped and Ti-doped crystals, respectively. The numbers 1,2 and 3,4 indicate the Fe atoms where the polaron is localized in the initial and final states, respectively. In the undoped slab in panels, the dotted circle indicates the Fe site between the initial and final polaron positions. The Fe at this site is replaced by Ti for calculation of the doped crystal.*

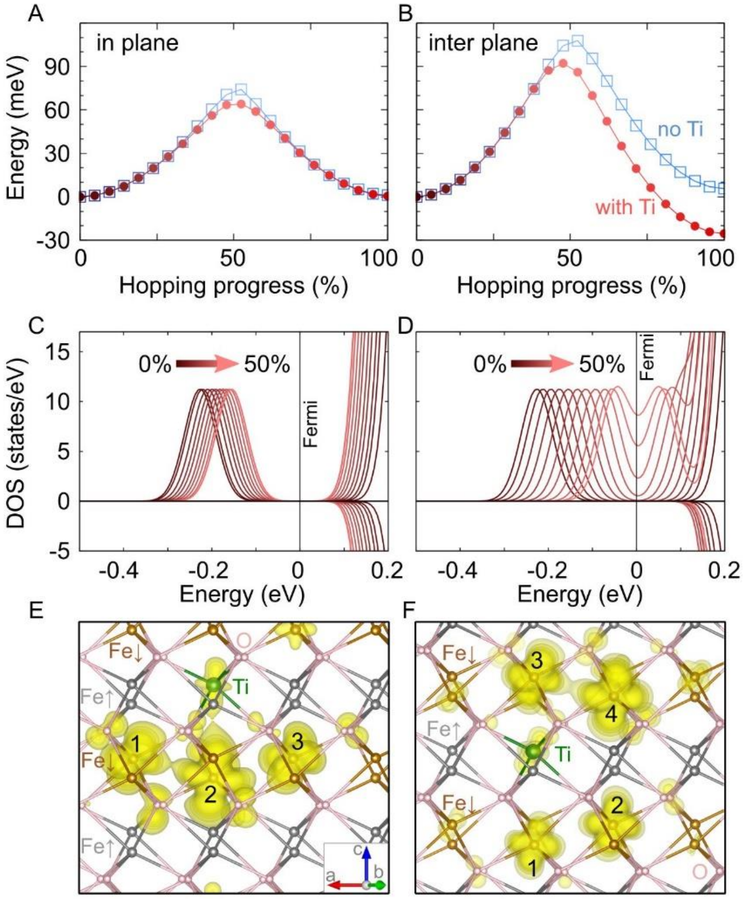


***Fig. S14. Electron-polaron hopping with and without the presence of Ti.*** *Panels A,C,E and B,D,F relate to the in-plane and inter-plane hopping, respectively. Panels A,B show the energy barriers as a function of the progressing lattice distortion without (blue) and with (red) Ti dopants. Panels C,D show the spin-resolved DOS during the hopping process (from 0% to 50% in gradient of red); only the case with the Ti dopant is shown. Panels E,F show the isodensity of the transition state (i.e., the polaron at 50% progress); O atoms are shown in pink, Ti in green, Fe in brown and grey to distinguish between opposite orientation of the local magnetic moments. In E, the polaron is hopping in-plane from the initial configuration at sites 1, 2 (0%) towards the final state at sites 2,3 (100%). F corresponds to an interplanar hop from sites 1,2 (0%) towards 3,4 (100%).*

***Table ST4: Site-resolved orbital occupation.*** *Projection of the polaronic state (obtained by DFT calculations in both the undoped and Ti-doped crystals) into the orbitals of the Fe sites 1, 2, 3, and 4, and the Fe/Ti site, between the initial and final polaron position (green, as labeled in Fig. S13). The values of 0% and 100% stand for the initial and final states, respectively.*

| | Site-resolved polaron occupation (%) | | | | |
|---|---|---|---|---|---|
| | Fe1 | Fe2 | Fe/Ti sub. site | Fe3 | Fe4 |
| Undoped, 0% hopping (Fig. S13a) | 40 | 40 | 0.2 | 0 | 0 |
| Undoped, 100% hopping (Fig. S13b) | 0 | 0 | 0.5 | 40 | 40 |
| Doped, 0% hopping (Fig. S13c) | 36 | 42 | 1.2 | 0 | 0 |
| Doped, 100% hopping (Fig. S13d) | 0 | 0 | 2.1 | 31 | 47 |

## 4. Additional supplementary files (captions)

**Movie S1:** Temperature-dependent evolution of a cloud of electron polarons, as simulated by Kinetic Monte Carlo. The data correspond to Figure 3G, *i.e.*, the model without a fixed lattice, the hop length of 2.5 nm, activation energy of 58 meV, and a prefactor of $10^{13}$ $s^{-1}$.

**Data S1: Other File Type_seq1_v1.rar**: Source codes to all KMC simulations.

**Data S2: Other File Type_seq2_v2.rar**: Coordinate files of the structures presented in the main text, as computed by DFT.

## REFERENCES AND NOTES


1. G. S. Parkinson, Iron oxide surfaces. *Surf. Sci. Rep.* **71**, 272–365 (2016).

2. K. Sivula, F. L. Formal, M. Grätzel, Solar water splitting: Progress using hematite (α-$Fe_2O_3$) photoelectrodes. *Chem. Sus. Chem.* **4**, 432–449 (2011).

3. J. Zhang, S. Eslava, Understanding charge transfer, defects and surface states at hematite photoanodes. *Sust. Energy Fuels* **3**, 1351–1364 (2019).

4. C. Franchini, M. Reticcioli, M. Setvin, U. Diebold, Polarons in materials. *Nat. Rev. Mater.* **6**, 560–586 (2021).

5. I. G. Austin, N. F. Mott, Polarons in crystalline and non-crystalline materials. *Adv. Phys.* **50**, 757–812 (2001).

6. V. Coropceanu, J. Cornil, D. A. S. Filho, Y. Olivier, R. Silbey, J.-L. Brédas, Charge transport in organic semiconductors. *Chem. Rev.* **107**, 926–952 (2007).

7. F. Ortmann, F. Bechstedt, K. Hannewald, Charge transport in organic crystals: Theory and modelling. *Phys. Stat. Solidi B* **248**, 511–525 (2011).

8. Y. Natanzon, A. Azulay, Y. Amouyal, Evaluation of polaron transport in solids from first-principles. *Israel J. Chem.* **60**, 768–786 (2020).

9. J. L. M. van Mechelen, D. van der Marel, C. Grimaldi, A. B. Kuzmenko, N. P. Armitage, N. Reyren, H. Hagemann, I. I. Mazin, Electron-phonon interaction and charge carrier mass enhancement in $SrTiO_3$. *Phys. Rev. Lett.* **100**, 226403 (2008).

10. C. D. Valentin, G. Pacchioni, A. Selloni, Reduced and n-Type doped $TiO_2$: Nature of $Ti^{3+}$ Species. *J. Phys. Chem. C* **113**, 20543–20552 (2009).

11. A. C. Papageorgiou, N. S. Beglitis, C. L. Pang, G. Teobaldi, G. Cabailh, Q. Chen, A. J. Fisher, W. A. Hofer, G. Thornton, Electron traps and their effect on the surface chemistry of $TiO_2$(110). *Proc. Natl. Acad. Sci. U.S.A.* **107**, 2391–2396 (2010).

12. J. M. D. Teresa, M. R. Ibarra, P. A. Algarabel, C. Ritter, C. Marquina, J. Blasco, J. García, A. Moral, Z. Arnold, Evidence for magnetic polarons in the magnetoresistive perovskites. *Nature* **386**, 256–259 (1997).

13. C. Jooss, L. Wu, T. Beetz, R. F. Klie, M. Beleggia, M. A. Schofield, S. Schramm, J. Hoffmann, Y. Zhu, Polaron melting and ordering as key mechanisms for colossal resistance effects in manganites. *Proc. Natl. Acad. Sci. U.S.A.* **104**, 13597–13602 (2007).

14. S. Song, J. Kim, D. Lee, J. Lee, T. Min, J.-A. Chae, J.-S. Bae, J. Lee, J.-S. Lee, S. Park, The effect of $Fe^{2+}$ state in electrical property variations of Sn-doped hematite powders. *J. Am. Ceram. Soc.* **100**, 3928–3934 (2017).

15. D. Cogan, L. A. Lonegran, Electrical conduction in $Fe_2O_3$ and $Cr_2O_3$. *Sol. State Commun.* **15**, 1517–1519 (1974).

16. T. J. Smart, A. C. Cardiel, F. Wu, K.-S. Choi, Y. Ping, Mechanistic insights of enhanced spin polaron conduction in CuO through atomic doping. *NPJ Computat. Mater.* **4**, 61 (2018).

17. R. Karsthof, M. Grundmann, A. M. Anton, F. Kremer, Polaronic interacceptor hopping transport in intrinsically doped nickel oxide. *Phys. Rev. B* **99**, 235201 (2019).

18. W. Zhang, F. Wu, J. Li, D. Yan, J. Tao, Y. Ping, M. Liu, Unconventional relation between charge transport and photocurrent via boosting small polaron hopping for photoelectrochemical water splitting. *ACS Energy Lett.* **3**, 2232–2239 (2018).

19. M. A. Henderson, Insights into the (1 × 1) to (2 × 1) phase transition of the α-$Fe_2O_3$(012) surface using EELS LEED and water TPD, *Surf. Sci.* **515**, 253–262 (2002).

20. F. Kraushofer, Z. Jakub, M. Bichler, J. Hulva, P. Drmota, M. Weinold, M. Schmid, M. Setvin, U. Diebold, P. Blaha, G. S. Parkinson, Atomic-scale structure of the hematite α-$Fe_2O_3$(1-102) "R-Cut" surface. *J. Phys. Chem. C* **122**, 1657–1669 (2018).

21. G. Franceschi, F. Kraushofer, M. Meier, G. S. Parkinson, M. Schmid, U. Diebold, M. Riva, A model system for photocatalysis: Ti-doped α-$Fe_2O_3(1\bar{1}02)$ single-crystalline films. *Chem. Mater.* **32**, 3753–3764 (2020).

22. A. G. Tamirat, J. Rick, A. A. Dubale, W.-N. Su, B.-J. Hwang, Using hematite for photoelectrochemical water splitting: A review of current progress and challenges. *Nanoscale Horiz.* **1**, 243–267 (2016).

23. C. Li, Z. Luo, T. Wang, J. Gong, Surface, bulk, and interface: Rational design of hematite architecture toward efficient photo-electrochemical water splitting. *Adv. Mater.* **30**, e1707502 (2018).

24. F. J. Giessibl, The qPlus sensor, a powerful core for the atomic force microscope. *Rev. Sci. Instrum.* **90**, 011101 (2019).

25. S. Sadewasser, T. Glatzel, *Kelvin Probe Force Microscopy* (Springer-Verlag, 2012).

26. L. Gross, F. Mohn, P. Liljeroth, J. Repp, F. J. Giessibl, G. Meyer, Measuring the charge state of an adatom with noncontact atomic force microscopy. *Science* **324**, 1428–1431 (2009).

27. M. Setvin, J. Hulva, G. S. Parkinson, M. Schmid, U. Diebold, Electron transfer between anatase $TiO_2$ and an $O_2$ molecule directly observed by atomic force microscopy. *Proc. Natl. Acad. Sci. U.S.A.* **114**, E2556–E2562 (2017).

28. S. Fatayer, B. Schuler, W. Steurer, I. Scivetti, J. Repp, L. Gross, M. Persson, G. Meyer, Reorganization energy upon charging a single molecule on an insulator measured by atomic force microscopy. *Nat. Nanotechnol.* **13**, 376–380 (2018).

29. C. S. Ahart, K. M. Rosso, J. Blumberger, Electron and hole mobilities in bulk hematite from spin-constrained density functional theory. *J. Am. Chem. Soc.* **144**, 4623–4632 (2022).

30. N. Iordanova, M. Dupuis, K. M. Rosso, Charge transport in metal oxides: A theoretical study of hematite α-$Fe_2O_3$. *J. Chem. Phys.* **122**, 144305 (2005).

31. M. Chen, A. C. Grieder, T. J. Smart, K. Mayford, S. McNair, A. Pinongcos, S. Eisenberg, F. Bridges, Y. Li, Y. Ping, The impacts of dopants on the small polaron mobility and conductivity in hematite–the role of disorder. *Nanoscale* **15**, 1619–1628 (2023).

32. C. Cheng, Y. Zhu, Z. Zhou, R. Long, W.-H. Fang, Photoinduced small electron polarons generation and recombination in hematite. *NPJ Computat. Mater.* **8**, 148 (2022).

33. A. V. Barzykin, M. Tachiya, Mechanism of charge recombination in dye-sensitized nanocrystalline semiconductors: random flight model. *J. Phys. Chem. B* **106**, 4356–4363 (2002).

34. J. Nelson, S. A. Haque, D. R. Klug, J. R. Durrant, Trap-limited recombination in dye-sensitized nanocrystalline metal oxide electrodes. *Phys. Rev. B* **63**, 205321 (2001).

35. F. Mortreuil, L. Boudou, K. Makasheva, G. Teyssedre, C. Villeneuve-Faure, Influence of dielectric layer thickness on charge injection, accumulation and transport phenomena in thin silicon oxynitride layers: A nanoscale study. *Nanotechnology* **32**, 065706 (2021).

36. D. Benjelloun, J. P. Bonnet, J. P. Doumerc, J. C. Launey, M. Onillon, Anisotropy in the electrical properties of zirconium-doped a-$Fe_2O_3$ single crystals. *Mat. Chem. Phys.* **20**, 1–12 (1988).

37. T. Nakau, Electrical conductivity of a-$Fe_2O_3$. *J. Phys. Soc. Jap.* **15**, 727 (1960).

38. C. M. Tian, W.-W. Li, Y. M. Lin, Z. Z. Yang, L. Wang, Y. G. Du, H. Y. Xiao, L. Qiao, J. Y. Zhang, L. Chen, D.-C. Qi, J. L. MacManus-Driscoll, K. H. L. Zhang, Electronic structure, optical properties, and photoelectrochemical activity of Sn-doped $Fe_2O_3$ thin films. *J. Phys. Chem. C* **124**, 12548–12558 (2020).

39. C. Sanchez, K. Sieber, G. Somorjai, The photoelectrochemistry of niobium doped α-$Fe_2O_3$. *J. Electroanal. Chem. Interfacial Electrochem.* **252**, 269–290 (1988).

40. Z. Zhou, R. Long, O. V. Prezhdo, Why silicon doping accelerates electron polaron diffusion in hematite. *J. Am. Chem. Soc.* **141**, 20222–20233 (2019).

41. D. Benjelloun, J.-P. Bonnet, P. Dordor, J.-C. Launay, M. Onillon, P. Hagenmuller, Anisotropie des propriétés électriques de monocristaux de $Fe_20_3$ dopés au nickel. *Rev. de Chemie Minerale* **21**, 781 (1984).

42. H. Liu, A. Wang, P. Zhang, C. Ma, C. Chen, Z. Liu, Y.-Q. Zhang, B. Feng, P. Cheng, J. Zhao, L. Chen, K. Wu, Atomic-scale manipulation of single-polaron in a two-dimensional semiconductor. *Nat. Commun.* **14**, 3690 (2023).

43. M. Cai, M.-P. Miao, Y. Liang, Z. Jiang, Z.-Y. Liu, W.-H. Zhang, X. Liao, L.-F. Zhu, D. West, S. Zhang, Y.-S. Fu, Manipulating single excess electrons in monolayer transition metal dihalide. *Nat. Commun.* **14**, 3691 (2023).

44. C. M. Yim, M. B. Watkins, M. J. Wolf, C. L. Pang, K. Hermansson, G. Thornton, Engineering polarons at a metal oxide surface. *Phys. Rev. Lett.* **117**, 116402 (2016).

45. M. Setvin, C. Franchini, X. Hao, M. Schmid, A. Janotti, M. Kaltak, C. G. V. d. Walle, G. Kresse, U. Diebold, A direct view at excess electrons in $TiO_2$ rutile and anatase. *Phys. Rev. Lett.* **113**, 086402 (2014).

46. S. Gerhold, M. Riva, B. Yildiz, M. Schmid, U. Diebold, Adjusting island density and morphology of the $SrTiO_3$(110)-(4 × 1) surface: Pulsed laser deposition combined with scanning tunneling microscopy. *Surf. Sci.* **651**, 76–83 (2016).

47. G. Franceschi, M. Schmid, U. Diebold, M. Riva, Reconstruction changes drive surface diffusion and determine the flatness of oxide surfaces. *J. Vac. Sci. Technol. A* **40**, 023206 (2022).

48. F. Huber, F. J. Giessibl, Low noise current preamplifier for qPlus sensor deflection signal detection in atomic force microscopy at room and low temperatures. *Rev. Sci. Instrum.* **88**, 073702 (2017).

49. W. Greiner, *Classical Electrodynamics* (Springer, 1998).

50. B. Nadler, U. Hollerbach, R. S. Eisenberg, Dielectric boundary force and its crucial role in gramicidin. *Phys. Rev. E* **68**, 021905 (2003).

51. G. Kresse, J. Furthmuller, Efficiency of ab-initio total energy calculations for metals and semiconductors using a plane-wave basis set. *Comput. Mater. Sci.* **6**, 15–50 (1996).

52. G. Kresse, D. Joubert, From ultrasoft pseudopotentials to the projector augmented-wave method. *Phys. Rev. E* **59**, 1758–1775 (1999).

53. A. V. Krukau, O. A. Vydrov, A. F. Izmaylov, G. E. Scuseria, Influence of the exchange screening parameter on the performance of screened hybrid functionals. *J. Chem. Phys.* **125**, 224106 (2006).

54. N. Adelstein, J. B. Neaton, M. Asta, L. C. D. Jonghe, Density functional theory based calculation of small-polaron mobility in hematite. *Phys. Rev. B* **89**, 245115 (2014).

55. T. J. Smart, Y. Ping, Effect of defects on the small polaron formation and transport properties of hematite from first-principles calculations. *J. Phys. Cond. Matt.* **29**, 394006 (2017).

56. C. S. Ahart, J. Blumberger, K. M. Rosso, Polaronic structure of excess electrons and holes for a series of bulk iron oxides. *Phys. Chem. Chem. Phys.* **22**, 10699–10709 (2020).

57. N. Ansari, K. Ulman, M. F. Camellone, N. Seriani, R. Gebauer, S. Piccinin, Hole localization in $Fe_2O_3$ from density functional theory and wave-function-based methods. *Phys. Rev. Mater.* **1**, 035404 (2017).

58. N. Deskins, M. Dupuis, Electron transport via polaron hopping in bulk TiO2: A density functional theory characterization. *Phys. Rev. E* **75**, 195212 (2007).

59. G. Franceschi, F. Kraushofer, M. Meier, G. S. Parkinson, M. Schmid, U. Diebold, M. Riva, A model system for photocatalysis: Ti-Doped $\alpha$-$Fe_2O_3$(1102) single-crystalline films. *Chem. Mater.* **32**, 3753–3764 (2020).

60. A. S. Lucier, H. Mortesen, Y. Sun, P. Grutter, Determination of the atomic structure of scanning probe microscopy tungsten tips by field ion microscopy. *Phys. Rev. B* **72**, 235420 (2005).

61. M. Setvin, J. Javorsky, D. Turcinkova, I. Matolinova, P. Sobotik, P. Kocan, I. Ostadal, Ultrasharp tungsten tips—Characterization and nondestructive cleaning. *Ultramicroscopy* **113**, 152–157 (2012).

62. M. N. O. Sadiku, R. C. Garcia, Monte Carlo floating random walk solution of Poisson's equation, in *Southeastcon 1993 Proceedings* (IEEE, 1993).

63. A. Kuznetsov, A. Sipin, Monte Carlo algorithms for the extracting of electrical capacitance. *Mathematics* **9**, 2922 (2021).

64. J. C. Papaioannou, G. S. Patermarakis, H. S. Karayianni, Electron hopping mechanism in hematite ($\alpha$-$Fe_2O_3$). *J. Phys. Chem. Sol.* **66**, 839–844 (2005).

65. M. Reticcioli, Z. Wang, M. Schmid, D. Wrana, L. A. Boatner, U. Diebold, M. Setvin, C. Franchini, Competing electronic states emerging on polar surfaces. *Nat. Commun.* **13**, 4311 (2022).

66. S. Giannini, J. Blumberger, Charge transport in organic semiconductors: The perspective from nonadiabatic molecular dynamics. *Acc. Chem. Res.* **55**, 819–830 (2022).

67. S. Onari, T. Arai, K. Kudo, Infrared lattice vibrations and dielectric dispersion in $\alpha-Fe_2O_3$. *Phys. Rev. B* **16**, 1717–1721 (1977).

68. R. A. Lunt, A. J. Jackson, A. Walsh, Dielectric response of $Fe_2O_3$ crystals and thin films. *Chem. Phys. Lett.* **586**, 67–69 (2013).

69. L. J. Lauhon, W. Ho, Direct observation of the quantum tunneling of single hydrogen atoms with a scanning tunneling microscope. *Phys. Rev. Lett.* **85**, 4566–4569 (2000).

70. C. T. Campbell, R. V. Sellers, Enthalpies and entropies of adsorption on well-defined oxide surfaces: experimental measurements. *Chem. Rev.* **113**, 4106–4135 (2013).

71. E. D. Grave, L. H. Bowen, D. D. Amarasiriwardena, R. E. Vandenberghe, $^{57}$Fe Mosbauer effect study of highly substituted aluminum hematites: Determination of the magnetic hyperfine field distributions. *J. Magnet. Magnetic Mater.* **72**, 129–140 (1988).

72. P. Sobotik, P. Kocan, I. Ostadal, Direct observation of Ag intercell hopping on the Si(111)-(7 x 7) surface. *Surf. Sci.* **537**, L442–446 (2003).

73. S. V. Divinski, A. Pokoev, N. Esakkiraja, A. Paul, A mystery of "sluggish diffusion" in high-entropy alloys: the truth or a myth? ArXiv:1804.03465 [cond-mat.mtrl-sci] (2018).

74. M. Kluge, H. R. Schober, Diffusion and jump-length distribution in liquid and amorphous Cu33Zr67. *Phys. Rev. B* **70**, 224209 (2004).
